# Metabolically Unhealthy Obesity: Novel Subtypes Defined by Integrated Metabolomics and Hematology

**DOI:** 10.3390/biomedicines14102358

**Published:** 2026-10-16

**Authors:** Xuan Huang, Yuhong Liu, Xiaoqian Zhu, Zhennan Lin, Yansong Guo, Kaiyang Lin

**Affiliations:** 1 Department of Cardiology, Shengli Clinical Medical College of Fujian Medical University, Fujian Provincial Hospital, Fuzhou University Affiliated Provincial Hospital, Fuzhou 355000, China; 2 Fujian Provincial Key Laboratory of Cardiovascular Disease, Fujian Provincial Center for Geriatrics, Fujian Provincial Clinical Research Center for Severe Acute Cardiovascular Diseases, Fuzhou 355000, China; 3 Fujian Heart Failure Center Alliance, Fuzhou 355000, China; 4 Center for Cardiovascular Epidemiology Research and Prevention of Fujian Provincial Hospital, Fuzhou 355000, China

**Keywords:** metabolomics, metabolically unhealthy obesity, cardiovascular–kidney–metabolic syndrome, risk stratification

## Abstract

**Objectives**: The aim was to identify metabolic subtypes within metabolically unhealthy obesity (MUO) using integrated metabolomics and hematology data and evaluate their differential risks for cardiovascular–kidney–metabolic (CKM)-related outcomes. **Methods**: Among 62,341 obese UK Biobank participants (43,577 MUO; 18,764 non-MUO), least absolute shrinkage and selection operator regression selected 67 biomarkers from 96 metabolites and serological parameters, followed by K-means clustering. Cox proportional hazards models assessed subtype-specific associations with incident CKM-related outcomes over a median 14.81-year follow-up. **Results**: Four metabolic subtypes were identified within MUO, characterized by distinct amino acid, renal, and lipid profiles. Subtype-specific associations with CKM-related outcomes differed substantially. Compared with non-MUO participants, Subtype II and Subtype IV were associated with higher coronary heart disease risk (HR, 1.34; 95% CI, 1.11–1.61 and HR, 1.37; 95% CI, 1.15–1.64, respectively), Subtype IV was associated with lower atrial fibrillation risk (HR, 0.73; 95% CI, 0.60–0.90), Subtype III with higher heart failure risk (HR, 1.42; 95% CI, 1.10–1.82), and Subtype I with higher chronic kidney disease risk (HR, 1.52; 95% CI, 1.22–1.91). **Conclusions**: Integrated metabolomic and hematologic profiling identified variation in the metabolic profiles of individuals with MUO. The four subtypes showed different patterns of association with CKM-related outcomes, suggesting that MUO encompasses diverse metabolic profiles that may not be fully captured by conventional metabolic syndrome criteria.

## 1. Introduction

Metabolically unhealthy obesity (MUO), defined by the co-occurrence of obesity and metabolic syndrome (MetS), represents a high-risk phenotype within the cardiovascular–kidney–metabolic (CKM) syndrome continuum, with established associations with cardiovascular disease, type 2 diabetes, chronic kidney disease, and premature mortality [1,2,3]. Current diagnostic frameworks rely on categorical MetS criteria—typically the presence of two or more cardiometabolic abnormalities—which reduce a continuous, multidimensional metabolic derangement into a binary label [4,5]. This dichotomous approach aggregates individuals with divergent pathophysiological profiles into a single risk stratum, failing to capture the varying severity of individual components or to recognize that specific combinations confer differential residual risks [6].

The heterogeneity of obesity and metabolic dysregulation is increasingly recognized, with individuals exhibiting divergent cardiometabolic risks despite comparable body mass index (BMI) [7,8]. Unsupervised machine learning approaches have identified reproducible obesity subtypes with distinct complication patterns [9,10]; however, these efforts have predominantly focused on broad obesity populations or the binary classification of metabolically healthy versus unhealthy obesity [9,10,11], leaving the internal heterogeneity of MUO unexplored. Recent data-driven cluster analyses leveraging metabolomic data have identified distinct obesity subtypes with markedly divergent cardiometabolic risks despite comparable anthropometric measures, underscoring the added predictive value of molecular phenotyping beyond BMI and waist circumference(WC) [12]. However, the resulting subtypes primarily reflect differences between broad metabolic health states rather than the nuanced pathophysiological variations within the metabolically unhealthy population itself. The present study addresses this gap by integrating NMR-based metabolomics with hematologic biomarkers to systematically define distinct metabolic subtypes within MUO and evaluate their differential risks for incident CKM-related outcomes.

Although the prognostic association between MUO and adverse CKM outcomes is well established [13,14,15], it remains unknown whether distinct metabolic subtypes within MUO exhibit differential risks for specific CKM-related events. To address this gap, this study leveraged the UK Biobank to perform robust clustering on a comprehensive panel of 96 metabolites and serological biomarkers within a well-defined MUO sub-cohort. Our primary objectives were to identify novel, metabolomics-driven subtypes and to rigorously evaluate their independent associations with major CKM-related outcomes and all-cause mortality, thereby advancing personalized risk prediction and prevention for this high-risk population.

## 2. Materials and Methods

### 2.1. Study Design and Population

This study utilized data from the UK Biobank, a prospective cohort that recruited over 500,000 individuals aged 40–69 years between 2006 and 2010 across the United Kingdom [16]. Participants attended baseline assessment centers in England, Scotland, and Wales, where they provided electronic informed consent. Baseline data on sociodemographic characteristics, lifestyle, environmental factors, and health status were collected via touch-screen questionnaires and nurse-led interviews. Physical measurements were taken, and biological samples were obtained. Medication use was recorded using Anatomical Therapeutic Chemical (ATC) codes. All participants completed informed consent, and ethical approval was obtained by the UK National Health Service (NHS) and National Research Ethics Service for the UK (No. 11/NW/0382).

After excluding participants with missing metabolomic or key laboratory data and those with a BMI < 30 kg/m^2^, 62,341 individuals were included and randomly split into a 70% training set (*n* = 43,639) and a 30% validation set (*n* = 18,702) (Supplemental Figure S1). The overall obese cohort was randomly split into training and validation sets; MUO participants within these sets were subsequently used for subtype discovery and validation.

### 2.2. Definition of Metabolically Unhealthy Obesity

MUO was defined as obesity (BMI ≥ 30 kg/m^2^) accompanied by MetS, based on an adapted version of the 2009 Harmonized Criteria [4]. MetS was diagnosed by the presence of at least three of five components: elevated WC, elevated triglycerides, elevated blood pressure or use of antihypertensives, elevated blood glucose or use of glucose-lowering medications, and reduced high-density lipoprotein cholesterol ((HDL-C) or use of lipid-modifying medications. Data on circulating glucose levels were obtained predominantly from non-fasting blood samples, which are more likely to be affected by recent food intake (compared to fasting samples), which can lead to high variability in glucose measurements. Therefore, we used glycated hemoglobin (HbA1c) as a proxy measure of glucose, based on the recommendations of the American Diabetes Association, with a cut point of HbA1c ≥ 5.7% (39 mmol/mol) to represent hyperglycemia [17]. Additionally, lipid-modifying medications are known to have multiple effects on the lipid profile (including HDL cholesterol and triglycerides) [18]. Thus, to prevent double counting, we assigned these medications to the reduced HDL-C group in our analysis. Medication usage was captured using ATC codes, informed by a thorough literature review of previous studies defining MetS using ATC codes [19,20,21,22,23,24,25,26,27,28,29], and with expert clinical advice. Detailed definitions of each component are provided in Supplemental Tables S1 and S2 [17,18,30].

### 2.3. Outcome Definitions

Incident CKM-related outcomes [31], including coronary heart disease (CHD), atrial fibrillation (AF), heart failure (HF), stroke, peripheral artery disease (PAD), and chronic kidney disease (CKD), and all-cause mortality were ascertained from hospital inpatient records and death registries in England, Scotland, and Wales. The ischemic and hemorrhagic stroke analyses were prespecified secondary outcomes, given their distinct pathophysiologies within the CKM framework. Diagnoses were defined using the International Classification of Diseases, Tenth Revision (ICD-10) codes, as specified in Supplemental Table S3.

### 2.4. Laboratory Data and Metabolomic Profiling

From the UK Biobank’s NMR metabolomic data (Class 220), we selected 64 metabolic biomarkers (Supplemental Table S4) after excluding HbA1c, HDL-C, triglycerides (TGs), all lipoprotein lipid subfraction concentrations, particle size measures, lipid-to-total-lipid percentages, apolipoprotein ratios, and fatty acid profiles and ratios. A further 38 hematological and serological markers relevant to obesity and MetS were obtained from baseline blood samples (Supplemental Table S5). HbA1c, HDL-C, and TG were not included as candidate clustering biomarkers because these measures were directly incorporated into the operational definition of MetS. Thus, 102 biomarker measurements were initially considered across the two data sources. After identifying overlapping biomarkers between the NMR metabolomic and hematological/serological datasets, including albumin, apolipoprotein B, creatinine, glucose, low density lipoprotein (LDL) cholesterol, and total cholesterol, the NMR-derived measurement was preferentially retained to avoid redundancy. This resulted in a final panel of 96 unique candidate biomarkers for subsequent feature selection.

### 2.5. Statistical Analysis

To identify biomarkers discriminating MUO from non-MUO status, we first performed Least Absolute Shrinkage and Selection Operator (LASSO) logistic regression in the training set, with MUO status as the dependent variable. The penalty parameter was selected by 5-fold cross-validation using the area under the receiver operating characteristic curve (AUC) as the performance measure, and the one-standard-error rule (λ1se) was used to obtain a parsimonious feature set. This procedure identified 67 non-zero biomarkers. The discriminative performance of the selected feature set was evaluated by the AUC in both the training and independent testing sets (Supplemental Figure S2). Among individuals classified as MUO in the training set, principal component analysis (PCA) was subsequently performed on the 67 selected biomarkers after centering and scaling. PC1 and PC2, explaining 19.19% and 7.40% of the total variance, respectively (26.59% cumulatively), were retained as a parsimonious representation of the dominant metabolic variation based on the scree plot and cluster separation, with higher-dimensional PCA solutions evaluated in sensitivity analyses (Supplemental Figure S5). Sensitivity analyses using higher-dimensional PCA representations were performed to assess the robustness of this choice (Supplemental Table S6). K-means clustering was then applied to the PC1–PC2 coordinates of the 30,533 MUO participants in the training set, using Euclidean distance with 25 random starts. The optimal number of clusters was determined using the elbow method, evaluating within-cluster sum of squared errors for k = 2 to 10, together with the silhouette coefficient (Supplemental Figure S3). Resampling-based reproducibility was assessed, and agreement between alternative clustering specifications was quantified using the adjusted Rand index (ARI) and normalized mutual information (NMI). For assignment in the independent testing set, each participant was allocated to the nearest training-derived cluster centroid based on Euclidean distance in the PC1–PC2 space.

Associations between MUO subtypes and incident outcomes were evaluated using Cox proportional hazards models with the non-MUO group as the reference. Model 1 was unadjusted. Model 2 was adjusted for age, sex, ethnicity, smoking status, and alcohol consumption. Model 3 additionally adjusted for systolic blood pressure, use of antihypertensive, glucose-lowering, and lipid-lowering medications, Townsend Deprivation Index, education level, and physical activity. The proportional hazards assumption was verified using Schoenfeld residuals. Kaplan–Meier curves were generated for cumulative incidence. The Benjamini–Hochberg procedure was applied across the 36 primary subtype–outcome comparisons to control the false discovery rate at P_FDR_ < 0.05 (Supplemental Table S10). In addition, within the MUO population, Cox proportional hazards models were fitted using Subtype IV as the reference group to directly compare the hazards of each other MUO subtype with Subtype IV (Supplemental Table S11). Global likelihood ratio tests were performed to assess overall heterogeneity across the four MUO subtypes for each clinical outcome, comparing fully adjusted models with and without the subtype variable. Pairwise Cox proportional hazards models were additionally fitted for all six subtype pairs for each of the nine outcomes using the same outcome-specific disease-free populations and Model 3 covariates (Supplemental Table S12). Fine–Gray subdistribution hazard models were used in sensitivity analyses to account for competing events (Supplemental Table S13). Potential effect modification by MetS components and sex was assessed using likelihood ratio tests of Subtype × MetS component and Subtype × Sex interaction terms, respectively (Supplemental Tables S14 and S15), with sex-stratified analyses performed to characterize associations separately in women and men. All analyses were performed using R (version 4.1.3). Detailed statistical methods, including sensitivity analyses and within-MUO subtype comparisons, are provided in the Supplemental Methods.

## 3. Results

### 3.1. Key Phenotypic and Metabolic Features of the Identified Subtypes

From the UK Biobank cohort of 502,401 individuals, 62,341 participants with BMI ≥ 30 kg/m^2^ were included after applying exclusion criteria (Supplemental Figure S1). Of these, 43,577 met the diagnostic criteria for MetS and were randomly assigned to a training set (*n* = 30,533, 70%) for subtype discovery and a validation set (*n* = 13,044, 30%) for confirmation, while the remaining 18,764 non-MUO individuals served as the reference group. To identify distinct subgroups within metabolically unhealthy obesity, we first employed LASSO regression to select the most discriminative metabolic features. Among the 96 metabolites and hematologic biomarkers that passed quality control, 67 features were identified as key predictors of metabolic syndrome status, with the final model achieving robust discrimination in both the training set (AUC = 0.868, 95% CI 0.864−0.871) and the validation set (AUC = 0.866, 95% CI 0.860−0.871) (Supplemental Figure S2).

**Figure 1:**
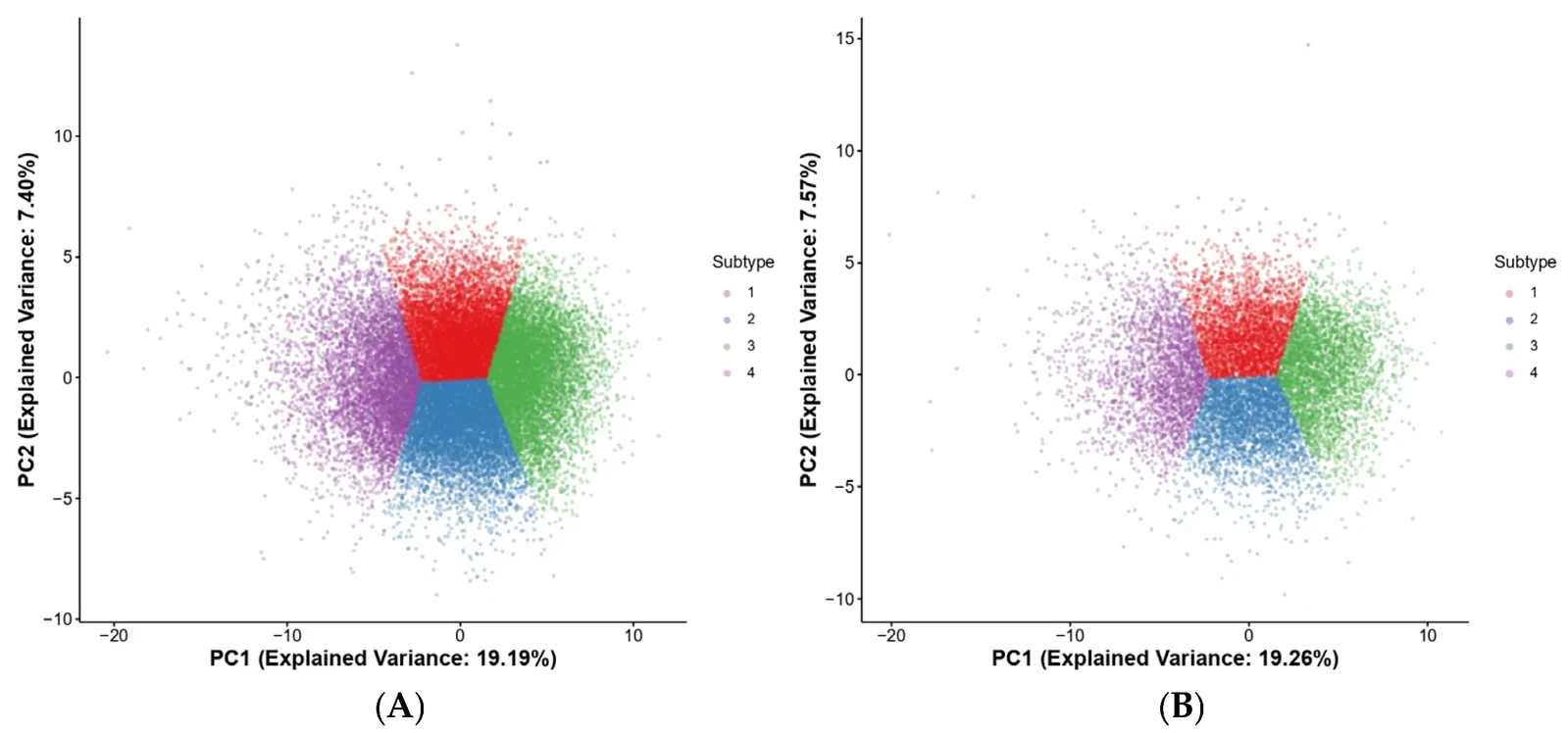
Distribution of MUO Subtypes by Principal Component in the Training and Validation Sets. (**A**): PCA plot of MUO individuals in the training set (*n* = 30,533); (**B**): PCA plot of MUO individuals in the validation set (*n* = 13,044). Abbreviations: PCA, principal component analysis; PC, principal component.

Principal component analysis was performed on these 67 selected features. The first two principal components accounted for 19.19% and 7.40% of the total variance, respectively (cumulative variance explained: 26.59%; Figure 1). Using PC1 and PC2, K-means clustering analysis was conducted on the 30,533 MUO individuals in the training set. Four clusters was the optimal number based on the elbow method and silhouette coefficient plots (Supplemental Figure S3). In the training set, the four subtypes comprised 8247 (27.01%), 7950 (26.04%), 8176 (26.78%), and 6160 (20.17%) individuals for Subtypes I through IV, respectively. The resulting clustering demonstrated high reproducibility across 100 resampling iterations, with a mean NMI of 0.944 and a mean ARI of 0.961.

### 3.2. Baseline Characteristics of the Novel Metabolic Subtypes

The demographic and clinical characteristics of the study population are summarized in Table 1 and Supplemental Table S9. Although many variables differed statistically across groups, the magnitude of between-group differences varied, with standardized mean difference (SMD) ranging from 0.119 to 1.574. Compared with Non-MUO participants, all four subtypes were older and exhibited more adverse cardiometabolic profiles, yet distinct patterns emerged across subtypes. Subtype I was predominantly male (82.6%), had the highest WC (110.56 cm), and showed low HDL-C (1.07 mmol/L) together with elevated triglycerides (2.98 mmol/L) and HbA1c (41.03 mmol/mol). Subtype II exhibited a striking female predominance (87.6% women), the highest HDL-C (1.37 mmol/L) among the MUO groups, and relatively lower triglycerides (1.90 mmol/L) and HbA1c (38.58 mmol/mol), consistent with a more favourable lipid phenotype. Subtype III was the oldest (59.14 years) and had the highest HbA1c (42.22 mmol/mol) and BMI (34.63 kg/m^2^), the lowest triglycerides (1.87 mmol/L), low HDL-C (1.10 mmol/L), and elevated WC (109.95 cm); men comprised 68.0% of this cluster. Subtype IV had the highest blood pressure (systolic blood pressure (SBP) 147.39 mmHg, diastolic blood pressure (DBP) 88.18 mmHg) and the highest triglycerides (3.09 mmol/L).

### 3.3. Metabolic Profiles of Novel Metabolic Subtypes

**Figure 2:**
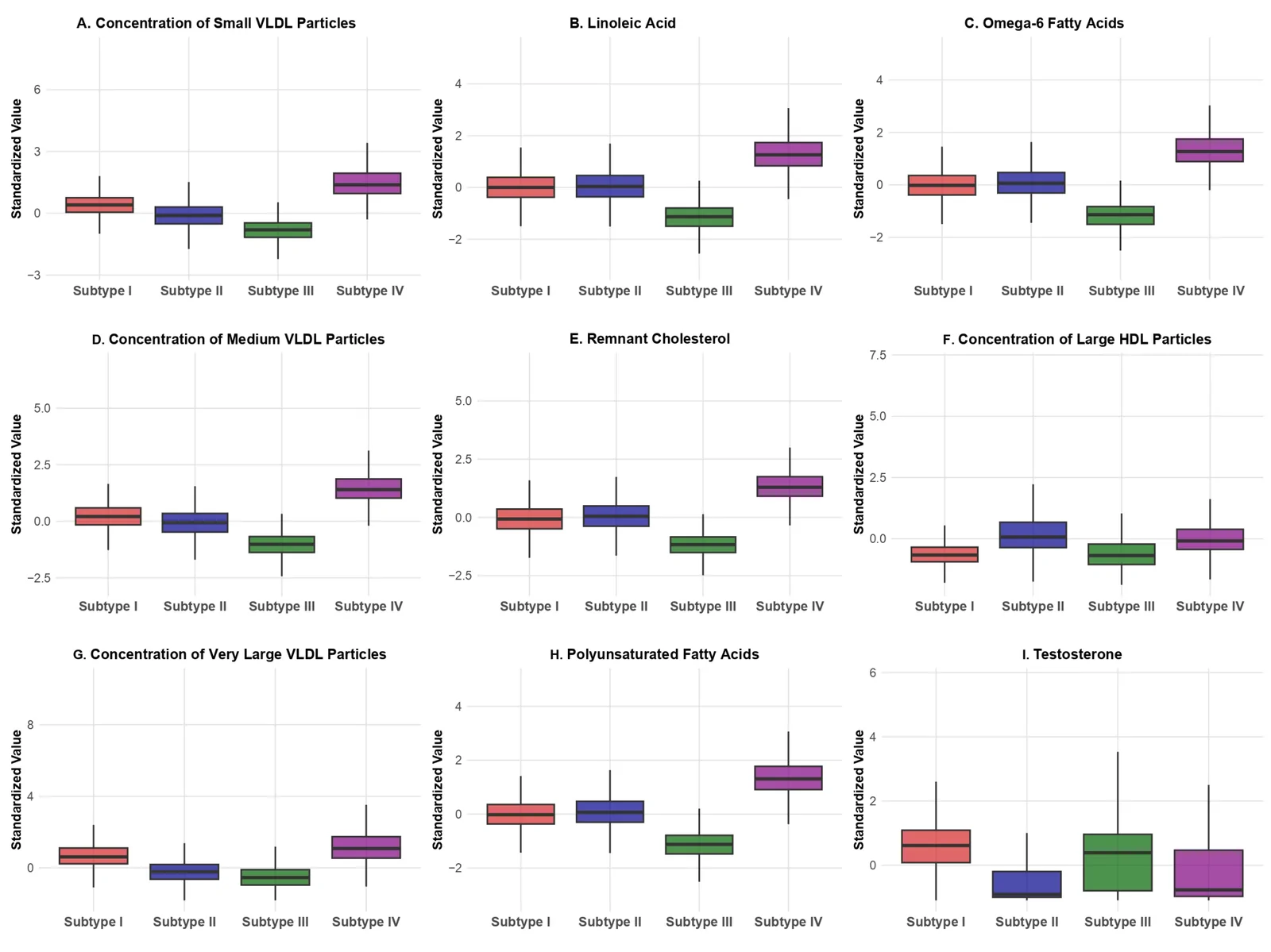
Top Nine Discriminative Metabolites Across the Four Metabolic Subtypes. (**A**): Concentration of Small VLDL Particles; (**B**): Linoleic Acid; (**C**): Omega-6 Fatty Acids; (**D**): Concentration of Medium VLDL Particles; (**E**): Remnant Cholesterol; (**F**): Concentration of Large HDL Particles; (**G**): Concentration of Very Large VLDL Particles; (**H**): Polyunsaturated Fatty Acids; (**I**): Testosterone. Notes: All metabolites were standardized to a mean of 0 and standard deviation of 1 within the validation set. Each box plot displays the distribution of standardized (z-score) metabolite levels across the four metabolic subtypes in the validation set. The horizontal line within each box represents the median; the lower and upper box boundaries represent the 25th and 75th percentiles (interquartile range, IQR), respectively; the whiskers extend to the most extreme values within 1.5 × IQR from the box edges. Significance was determined by one-way ANOVA with FDR correction (Supplemental Table S9). Abbreviations: VLDL, Very-Low-Density Lipoprotein; HDL, High-Density Lipoprotein.

The metabolic characterization of the four subtypes was first established in the validation set (*n* = 13,044). A total of 66 of 67 metabolites differed significantly across the four clusters (P_FDR_ < 0.05 for 66 of 67 metabolites; Supplemental Figure S4 and Supplemental Table S8), underscoring the robustness of the metabolic separation. The nine most discriminative features, including linoleic acid(LA), remnant cholesterol, omega-6 fatty acids, polyunsaturated fatty acids, testosterone, and lipoprotein subclass concentrations, define subtype-specific metabolic signatures (Figure 2). Based on their characteristic metabolite patterns, we designated the subtypes as follows:

Subtype I (amino acid-enriched and renal stress, *n* = 3442) was characterized by elevated levels of several amino acids, including leucine, alanine, tyrosine, phenylalanine, and histidine, together with higher creatinine, urate, cystatin C, and urea, a pattern suggestive of renal stress and altered amino acid metabolism.

Subtype II (metabolically preserved, *n* = 3412) displayed a relatively favourable metabolic profile, with the highest apolipoprotein A and large HDL particles, along with preserved medium and very large HDL particles. It also had higher sex hormone binding globulin (SHBG) and lower testosterone, urate, creatinine, leucine, haemoglobin, and haematocrit, with only modestly elevated C-reactive protein (CRP), consistent with preserved HDL function and minimal metabolic disturbance.

Subtype III (lipid-depleted, *n* = 3584) exhibited broad suppression of lipid pathways, including markedly lower LA, omega-6 and polyunsaturated fatty acids, total fatty acids, and multiple very low-density lipoprotein (VLDL), LDL, and (intermediate-density lipoprotein) IDL subclasses, as well as reduced total free cholesterol and remnant cholesterol. HDL-related measures were also low.

Subtype IV (lipid-enriched, *n* = 2606) showed widespread upregulation of lipid species, including LA, omega-6 and polyunsaturated fatty acids, saturated and monounsaturated fatty acids, remnant cholesterol, total free cholesterol, and multiple VLDL and LDL subfractions, together with higher docosahexaenoic acid (DHA), glycoprotein acetyls, and very large HDL particles, indicating a lipid-enriched subtype with prominent atherogenic dyslipidaemia.

### 3.4. Long-Term Survival Outcomes and Cardiovascular–Kidney–Metabolic-Related Event Incidence Risks by Subtypes

**Figure 3:**
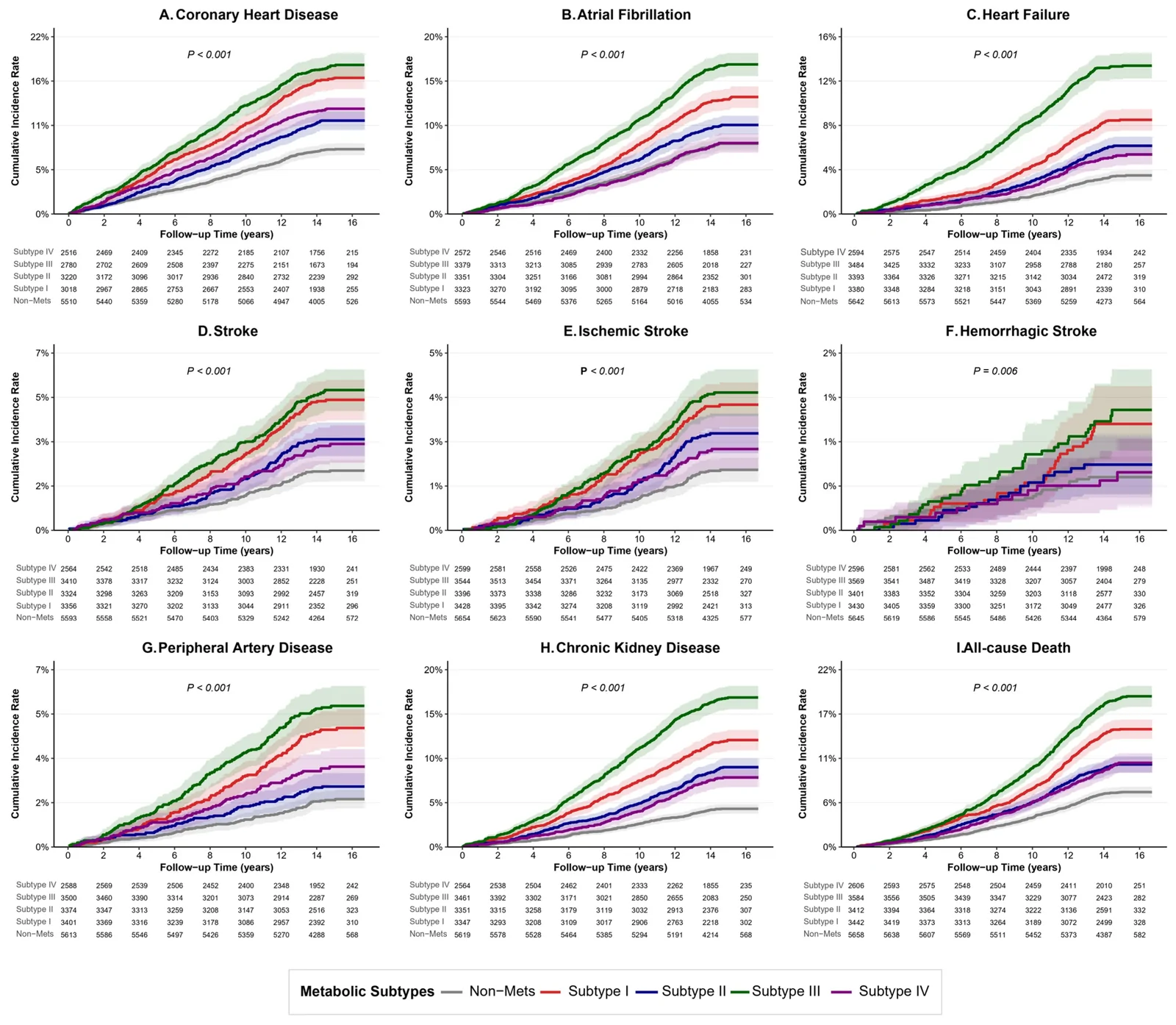
Cumulative Incidence of Cardiovascular–Kidney–Metabolic-related Outcomes by Metabolic Subtype. (**A**): Coronary Heart Disease; (**B**): Atrial Fibrillation; (**C**): Heart Failure; (**D**): Stroke; (**E**): Ischemic Stroke; (**F**): Hemorrhagic Stroke; (**G**): Peripheral Artery Disease; (**H**): Chronic Kidney Disease; (**I**): All-cause Death. Notes: The Kaplan–Meier survival curve was employed to estimate the cumulative density of events at specific time points. The Log-rank test was used to evaluate statistical differences between survival curves. Significance level: *p* < 0.05.

In the validation set (*n* = 13,044), we assessed the association of the four metabolic subtypes with the risk of multiple outcomes, including CHD, AF, HF, stroke (including ischemic stroke and hemorrhagic stroke), PAD, CKD, and all-cause mortality. With a median follow-up of 14.81 years (Q1–Q3: 13.95–15.58), Kaplan–Meier curves showed marked differences in the cumulative incidence of these events among the four metabolic subtypes and non-MUO individuals (Figure 3). In unadjusted analyses, Subtype III (Lipid-depleted) displayed the highest event rates for most outcomes, followed by Subtype I (Amino acid-enriched and renal stress), whereas Subtypes II (Metabolically preserved) and IV (Lipid-enriched) generally exhibited lower risks, with Subtype IV often demonstrating the weakest or non-significant associations. After multivariable adjustment, the hazard ratios showed subtype-specific differences in their associations with clinical outcomes (Table 2; Supplemental Table S10). Compared with non-MUO individuals, results for MUO individuals were as follows:

CHD: Subtype IV showed a positive association with CHD (HR 1.37, 95% CI 1.15–1.64, *p* < 0.001; P_FDR_ = 0.011), followed by Subtype II (HR 1.34, 95% CI 1.11–1.61, *p* = 0.002; P_FDR_ = 0.024); both associations remained significant after FDR correction. Subtype III was nominally associated with CHD (HR 1.21, 95% CI 1.01–1.46, *p* = 0.042) but did not survive FDR correction (P_FDR_ = 0.180), whereas Subtype I was not significantly associated (HR 1.15, 95% CI 0.97–1.37, *p* = 0.104; P_FDR_ = 0.288).

Atrial fibrillation: Subtype IV was inversely associated with AF (HR 0.73, 95% CI 0.60–0.90, *p* = 0.003; P_FDR_ = 0.027), which remained significant after FDR correction. Subtypes I, II, and III showed no significant associations (all *p* > 0.05; all P_FDR_ > 0.05).

Heart failure: Subtype III was associated with elevated HF risk (HR 1.42, 95% CI 1.10–1.82, *p* = 0.006; P_FDR_ = 0.046) and remained significant after FDR correction; Subtypes I, II, and IV were not significantly associated.

CKD: Subtype I showed a positive association with CKD (HR 1.52, 95% CI 1.22–1.91, *p* < 0.001; P_FDR_ = 0.009) and remained significant after FDR correction. Subtype IV (HR 1.33, 95% CI 1.05–1.69, *p* = 0.019; P_FDR_ = 0.117) and Subtype III (HR 1.27, 95% CI 1.00–1.60, *p* = 0.046; P_FDR_ = 0.180) were nominally associated but did not remain significant after FDR correction, while Subtype II was not significantly associated (HR 1.12, 95% CI 0.89–1.40, *p* = 0.351; P_FDR_ = 0.574).

All-cause mortality: Subtype IV was nominally associated with higher risk (HR 1.24, 95% CI 1.02–1.50, *p* = 0.034; P_FDR_ = 0.174), and Subtype II was borderline (HR 1.21, 95% CI 1.00–1.47, *p* = 0.050; P_FDR_ = 0.180); however, no subtype remained significant after FDR correction.

Total stroke, ischemic stroke, hemorrhagic stroke, and peripheral artery disease: None of the four subtypes showed statistically significant associations in the fully adjusted model (all *p* > 0.05; all P_FDR_ > 0.05). Overall, after FDR correction, significant associations persisted for CHD Subtypes II and IV, AF Subtype IV, HF Subtype III, and CKD Subtype I (Supplemental Table S10).

Sensitivity analyses within the MUO population using Subtype IV as the reference identified nominal associations of Subtype II with lower PAD risk and Subtypes II and III with higher risks of AF, as well as Subtype III with higher HF risk; none remained significant after FDR correction (Supplemental Table S11). Fully adjusted Fine–Gray models yielded results broadly consistent with the primary Cox analyses (Supplemental Table S13). In the validation set (*n* = 13,044), nominal Subtype × MetS component interactions were observed for AF with elevated triglycerides, hemorrhagic stroke with reduced HDL cholesterol, and all-cause mortality with elevated blood glucose (P-interaction = 0.024, 0.042, and 0.022, respectively), but none survived FDR correction (all P_FDR_ > 0.05; Supplemental Table S14). Sex-stratified analyses suggested potential heterogeneity, with stronger CHD associations for Subtypes II and III and a stronger CKD association for Subtype I in women; inverse associations of Subtypes II and III with hemorrhagic stroke were also observed in women but not men (Supplemental Table S15). No consistent sex-specific patterns were observed for the other outcomes.

## 4. Discussion

This study identified four distinct metabolic subtypes among individuals with obesity and MetS (Graphical Abstract), with different patterns of association with CKM-related outcomes. These findings provide a descriptive taxonomy of MUO heterogeneity based on integrated metabolomics and hematology, and an assessment of subtype-specific associations with CKM-related outcomes. Our core finding demonstrates that the clinical trajectory in MUO is not determined by the simple summation of individual risk factors, but rather by the integrated equilibrium of the entire metabolic network.

Graphical abstract. Integrated metabolomics and hematology profiling reveals four distinct metabolic subtypes of MUO with differential cardiovascular–kidney–metabolic-related outcomes. Abbreviations: LASSO, Least Absolute Shrinkage and Selection Operator; PC, Principal Component; ω-6, Omega-6 Fatty Acids; VLDL, Very-Low-Density Lipoprotein; LDL-C, Low-Density Lipoprotein Cholesterol; HbA1c, Glycated Hemoglobin; HDL-C, High-Density Lipoprotein Cholesterol; ApoA, Apolipoprotein A; BCAA, Branched-Chain Amino Acids; DHA, Docosahexaenoic Acid.

An important finding of this study was the variation in outcome associations across MUO subtypes. Subtype I, characterized by an amino acid-enriched and renal-stress profile, showed a positive association with CKD. Subtype III displayed a lipid-depleted profile and was positively associated with heart failure, whereas Subtype IV, characterized by a lipid-enriched profile, showed a positive association with CHD and an inverse association with atrial fibrillation. These findings suggest that individuals meeting the same MUO criteria may exhibit different patterns of metabolic characteristics and outcome associations, although the extent of between-subtype risk heterogeneity should be interpreted cautiously given the modest cluster separation and the observational nature of the analyses [32,33].

An important consideration is the marked difference in sex distribution across the metabolic subtypes, particularly the predominance of women in Subtype II and men in Subtype I. Notably, this sex distribution was accompanied by differences in sex-hormone-related biomarkers: Subtype I showed higher testosterone, whereas Subtype II showed higher SHBG and lower testosterone. Sex hormones and their binding proteins have been linked to adipose tissue distribution, insulin sensitivity, and lipid and glucose metabolism, with the direction and magnitude of these associations differing by sex [34,35,36]. Therefore, the observed differences in testosterone and SHBG may reflect underlying sex-related metabolic biology and could have contributed to the metabolic structure captured by the clustering analysis. However, these findings are observational and do not establish that sex hormones causally determined subtype formation. Although sex was adjusted for in the primary outcome models, such adjustment does not determine the extent to which sex-related biology contributed to subtype formation. We therefore performed sex-stratified analyses to examine whether associations between metabolic subtypes and clinical outcomes were consistent across women and men.

Subtype I showed a positive association with CKD and was characterized by renal function biomarkers, including higher levels of urea, urate, and creatinine. This pronounced renal vulnerability defines the central clinical feature of this metabolic phenotype. The distinctive metabolic signature of Subtype I, characterized by profound elevations in branched-chain amino acids (BCAAs; leucine, isoleucine, valine), increased very-large VLDL particles, elevated glycoprotein acetyls, and high levels of gluconeogenic amino acids (alanine, glycine), provides critical insight into the mechanisms underlying both renal and cardiovascular risk. A longitudinal cohort of US women with >18 years of follow-up demonstrated a significant positive association between baseline plasma levels of BCAAs and incident CVD events [37]. Thus, Subtype I exemplifies the value of integrating renal and metabolic profiling when stratifying cardiometabolic risk in MUO.

Subtype II suggests a metabolically less perturbed state with a prominent HDL-related signature. This discordance between an HDL-rich phenotype and increased CHD risk underscores the limitations of interpreting HDL abundance as a surrogate for cardiovascular protection. HDL and its major structural protein, apolipoprotein A-I, participate in cholesterol efflux, reverse cholesterol transport, endothelial signalling, and anti-inflammatory and antioxidative processes [38]. Nevertheless, extensive evidence indicates that the cardiovascular effects of HDL depend not only on circulating HDL-C concentration or particle abundance but also on particle composition and functional properties, including cholesterol efflux capacity and anti-inflammatory activity [39,40]. Importantly, the association of Subtype II with CHD should not be interpreted as evidence that HDL-rich metabolism is intrinsically harmful. Rather, it may indicate that HDL-related measures capture only one component of a broader metabolic phenotype and that the biological quality and function of HDL particles, together with other unmeasured determinants of coronary atherosclerosis, may be more relevant than HDL abundance alone. Longitudinal studies indicate that metabolically healthy obesity is frequently unstable, with a substantial proportion of individuals transitioning to metabolically unhealthy states over time and such transitions being associated with higher subsequent cardiovascular risk [41,42]. Accordingly, Subtype II may represent a relatively preserved metabolic phenotype at baseline while retaining measurable coronary vulnerability, highlighting the heterogeneity of MUO and the importance of considering HDL function and longitudinal metabolic trajectories rather than HDL concentration alone.

Subtype III exhibited broad suppression across circulating lipid pathways, encompassing multiple VLDL, LDL and IDL subclasses, remnant and free cholesterol, HDL-related measures, total fatty acids, and omega-6 polyunsaturated fatty acids (PUFAs), with particularly pronounced depletion of LA. Importantly, this metabolic pattern was associated with a higher hazard of HF after multivariable adjustment. Indeed, a recent meta-analysis of 52 observational studies including 93,286 patients with heart failure found that higher circulating concentrations of total cholesterol, HDL cholesterol, LDL cholesterol and triglycerides were generally associated with lower mortality, while lower total and LDL cholesterol remained associated with higher mortality after multivariable adjustment [43]. Concurrent with these metabolic disturbances, Subtype I exhibits profound deficits in PUFAs, particularly LA. In patients with acute decompensated heart failure, lower circulating n-6 PUFA concentrations were independently associated with a higher risk of death or worsening heart failure [44]. However, prospective population-based evidence linking circulating LA specifically to incident heart failure remains inconsistent [45], and therefore the present findings should be interpreted as an association rather than evidence of causality. The concomitant elevation of direct bilirubin provides an additional indication of systemic cardiohepatic stress. Direct bilirubin has been independently associated with mortality and heart-failure readmission in both acute decompensated heart failure and heart failure with preserved ejection fraction (HFpEF), including in a cohort of 19,837 patients with HFpEF [46]. Given the continuously high energetic demands of the myocardium, disturbances in lipid metabolism may compromise substrate utilization and myocardial energy homeostasis, while accumulation of lipotoxic lipid species may further link systemic metabolic stress to persistent myocardial injury and dysfunction [47]. Taken together, Subtype III may therefore represent a metabolically vulnerable phenotype in which broad depletion of circulating lipids and essential PUFAs coexists with cardiohepatic stress, providing a potential metabolic signature of increased heart failure susceptibility despite the absence of conventionally elevated lipid risk markers.

Subtype IV was characterized by a lipid-enriched profile, with higher levels of remnant cholesterol, total free cholesterol, multiple VLDL and LDL subclasses, and very-large HDL particles. As expected, given this markedly atherogenic lipid profile, this subtype exhibited a significantly elevated risk of CHD, confirming that the classic pro-atherogenic lipo-proteins are indeed driving coronary risk in this population. Interestingly, despite having the highest CHD risk, Subtype IV was inversely associated with incident AF. A meta-analysis of 11 cohort studies reported inverse associations of both total cholesterol and LDL cholesterol with incident AF, while a study similarly found that higher LDL cholesterol and total cholesterol were associated with lower AF incidence [48,49]. More recent UK Biobank data have extended these observations to remnant cholesterol, with lower rather than higher remnant cholesterol associated with greater incident AF risk [50]. Several mechanisms have been proposed, including effects of lipid availability on myocardial membrane composition and electrophysiological properties, but the biological basis of these associations remains incompletely understood. Importantly, these epidemiological observations do not imply that atherogenic lipoproteins are protective against AF, nor do they diminish their established role in atherosclerotic disease. Thus, Subtype IV may be viewed as the most distinctly atherogenic metabolic phenotype in this cohort, while its divergent association with AF illustrates that the clinical consequences of metabolic dysregulation cannot be inferred from a single cardiovascular endpoint.

Nevertheless, several limitations should be considered. The UK Biobank is predominantly of European ancestry, limiting generalisability to other populations; future validation in non-European cohorts should involve independent feature selection and de novo clustering rather than direct transfer of the European-derived model. Although we confirmed internal validity, our results would still benefit from external validation in a different population. The NMR metabolomics platform, while standardised, does not capture the full metabolome, and some biomarkers lack disease specificity. CKD was defined using hospitalisation records rather than estimated glomerular filtration rate (eGFR) or albuminuria measurements, potentially underestimating early-stage disease. Finally, the cost and limited accessibility of metabolomic profiling, together with the added complexity for primary care, present barriers to clinical translation. Prospective trials and implementation research are needed to determine whether subtype-guided management improves outcomes and is feasible in real-world settings.

## 5. Conclusions

In conclusion, this metabolomics-driven cluster analysis identified four metabolic subtypes among individuals with metabolically unhealthy obesity, with different patterns of association with cardiovascular–kidney–metabolic outcomes. Integrated metabolomic and hematologic profiling revealed variation in metabolic characteristics and outcome associations within MUO, including associations with CKD, heart failure, CHD, and atrial fibrillation. These findings suggest that MUO may encompass heterogeneous metabolic profiles that are not fully captured by conventional metabolic syndrome criteria.

## Figures and Tables

**Table 1:** Baseline Characteristics of the Study Population Stratified by Metabolic Subtypes.

Variable	Total Set					*p*-Value	SMD
	Non-MUO (N = 18,764)	Subtype I (N = 11,689)	Subtype II (N = 11,362)	Subtype III (N = 11,760)	Subtype IV (N = 8766)		
Age (years) (mean (SD))	54.78 (7.94)	57.10 (7.80)	57.61 (7.71)	59.14 (7.47)	56.99 (7.44)	<0.001	0.565
Sex (%)							
Female	10,954 (58.4%)	2034 (17.4%)	9952 (87.6%)	3760 (32.0%)	5688 (64.9%)	<0.001	0.845
Male	7810 (41.6%)	9655 (82.6%)	1410 (12.4%)	8000 (68.0%)	3078 (35.1%)	<0.001	0.845
SBP (mmHg) (mean (SD))	139.01 (19.55)	147.03 (17.79)	145.26 (18.08)	143.88 (17.93)	147.39 (18.44)	<0.001	0.441
DBP (mmHg) (mean (SD))	84.56 (10.34)	87.58 (10.28)	86.15 (9.90)	84.23 (10.46)	88.18 (10.09)	<0.001	0.355
WC (cm) (mean (SD))	99.82 (10.20)	110.56 (9.70)	102.65 (9.94)	109.95 (10.90)	104.72 (9.90)	<0.001	1.079
BMI (kg/m^2^) (median [IQR])	32.93 (3.22)	34.16 (3.84)	34.57 (4.23)	34.63 (4.31)	33.99 (3.67)	<0.001	0.446
Smoking status (%)							
Never	10,548 (56.5%)	5051 (43.5%)	6345 (56.2%)	5257 (45.0%)	4349 (50.0%)	<0.001	0.264
Former	6630 (35.5%)	5156 (44.4%)	4001 (35.4%)	5257 (45.0%)	3388 (38.9%)	<0.001	0.264
Current	1505 (8.1%)	1405 (12.1%)	946 (8.4%)	1160 (9.9%)	964 (11.1%)	<0.001	0.264
Alcohol drinker status (%)							
Never	820 (4.4%)	468 (4.0%)	902 (8.0%)	744 (6.3%)	459 (5.2%)	<0.001	0.172
Former	623 (3.3%)	578 (5.0%)	551 (4.9%)	722 (6.2%)	366 (4.2%)	<0.001	0.172
Current	17,277 (92.3%)	10,605 (91.0%)	9866 (87.2%)	10,253 (87.5%)	7921 (90.6%)	<0.001	0.172
Ethnic (%)							
White	16,468 (87.8%)	10,651 (91.1%)	9903 (87.2%)	10,263 (87.3%)	8008 (91.4%)	<0.001	0.119
Non-White	2280 (12.2%)	1023 (8.8%)	1440 (12.7%)	1486 (12.6%)	748 (8.5%)	<0.001	0.119
TG (mmol/L) (median [IQR])	1.48 (0.75)	2.98 (1.25)	1.90 (0.68)	1.87 (0.96)	3.09 (1.24)	<0.001	1.574
HDL-C (mmol/L) (median [IQR])	1.45 (0.31)	1.07 (0.20)	1.37 (0.29)	1.10 (0.24)	1.31 (0.27)	<0.001	1.459
HbA1c (mmol/mol) (median [IQR])	34.46 (3.45)	41.03 (11.75)	38.58 (7.03)	42.22 (10.89)	38.58 (8.08)	<0.001	0.961

Abbreviations: DBP, diastolic blood pressure; SBP, systolic blood pressure; WC, waist circumference; BMI, body mass index; HDL-C, high-density lipoprotein cholesterol; TG, triglycerides; HbA1c, glycated hemoglobin; MUO, metabolically unhealthy obesity; SMD, standardized mean difference. Notes: Means (SD) are reported for continuous variables; in contrast, count (%) is presented for categorical features. Continuous variables were compared using the Kruskal–Wallis rank-sum test, and categorical variables were analyzed with Pearson’s chi-squared test. *p*-values were used to assess statistical significance, whereas SMDs were used to describe the magnitude of between-group differences.

**Table 2:** Associations of Metabolic Subtypes with Incident Cardiovascular–Kidney–Metabolic Outcomes and All-Cause Mortality in the Validation Set.

				Model 1	Model 2	Model 3
Outcomes	Subtypes	Events	Rate per 1000 PY	HR (95% CI) *p*-Value	HR (95% CI) *p*-Value	HR (95% CI) *p*-Value
CHD	I	479	11.96	2.20 (1.93–2.51) <0.001	1.51 (1.32–1.73) <0.001	1.15 (0.97–1.37) 0.104
	II	352	8.00	1.47 (1.28–1.69) <0.001	1.52 (1.31–1.77) <0.001	1.34 (1.11–1.61) 0.002
	III	481	13.37	2.45 (2.15–2.79) <0.001	1.70 (1.48–1.94) <0.001	1.21 (1.01–1.46) 0.042
	IV	313	9.16	1.69 (1.46–1.95) <0.001	1.57 (1.35–1.81) <0.001	1.37 (1.15–1.64) <0.001
AF	I	416	9.29	1.70 (1.49–1.94) <0.001	1.16 (1.01–1.34) 0.036	0.91 (0.76–1.08) 0.275
	II	326	7.07	1.29 (1.12–1.49) <0.001	1.16 (1.00–1.34) 0.055	1.00 (0.83–1.20) 0.962
	III	540	12.29	2.25 (1.99–2.56) <0.001	1.43 (1.25–1.63) <0.001	0.97 (0.80–1.16) 0.723
	IV	198	5.51	1.00 (0.85–1.19) 0.977	0.87 (0.74–1.03) 0.108	0.73 (0.60–0.90) 0.003
HF	I	273	5.84	2.55 (2.12–3.07) <0.001	1.72 (1.42–2.09) <0.001	1.08 (0.84–1.38) 0.557
	II	200	4.18	1.82 (1.49–2.22) <0.001	1.55 (1.26–1.90) <0.001	1.13 (0.87–1.47) 0.375
	III	447	9.67	4.25 (3.59–5.04) <0.001	2.59 (2.17–3.10) <0.001	1.42 (1.10–1.82) 0.006
	IV	132	3.59	1.56 (1.25–1.95) <0.001	1.33 (1.06–1.67) 0.012	1.08 (0.82–1.42) 0.590
Stroke	I	155	3.32	2.21 (1.74–2.80) <0.001	1.62 (1.26–2.08) <0.001	1.17 (0.85–1.60) 0.344
	II	109	2.31	1.54 (1.19–1.99) 0.001	1.41 (1.08–1.85) 0.011	1.17 (0.84–1.63) 0.344
	III	166	3.58	2.38 (1.88–3.01) <0.001	1.66 (1.30–2.12) <0.001	1.06 (0.75–1.50) 0.736
	IV	79	2.17	1.44 (1.08–1.91) 0.012	1.29 (0.97–1.72) 0.077	0.89 (0.62–1.28) 0.519
Ischemic Stroke	I	116	2.43	2.11 (1.61–2.76) <0.001	1.50 (1.13–1.99) 0.005	1.10 (0.77–1.57) 0.608
	II	90	1.87	1.61 (1.21–2.15) 0.001	1.53 (1.13–2.06) 0.006	1.33 (0.92–1.93) 0.128
	III	129	2.67	2.32 (1.78–3.02) <0.001	1.56 (1.18–2.05) 0.002	1.01 (0.68–1.50) 0.953
	IV	58	1.57	1.35 (0.98–1.88) 0.069	1.22 (0.88–1.70) 0.233	0.91 (0.61–1.37) 0.663
Hemorrhagic Stroke	I	30	0.62	1.96 (1.16–3.32) 0.012	1.64 (0.93–2.87) 0.087	1.43 (0.73–2.81) 0.299
	II	19	0.39	1.23 (0.68–2.23) 0.487	0.94 (0.51–1.76) 0.856	0.59 (0.27–1.28) 0.178
	III	34	0.69	2.18 (1.31–3.64) 0.003	1.65 (0.96–2.82) 0.069	1.10 (0.53–2.27) 0.796
	IV	12	0.32	1.02 (0.51–2.02) 0.961	0.88 (0.44–1.75) 0.712	0.59 (0.25–1.40) 0.231
PAD	I	149	3.15	2.54 (1.97–3.27) <0.001	1.80 (1.38–2.35) <0.001	1.01 (0.72–1.42) 0.951
	II	77	1.61	1.29 (0.96–1.74) 0.089	1.14 (0.84–1.54) 0.414	0.70 (0.48–1.03) 0.069
	III	181	3.81	3.07 (2.41–3.91) <0.001	2.01 (1.56–2.59) <0.001	0.86 (0.60–1.23) 0.396
	IV	78	2.12	1.71 (1.27–2.30) <0.001	1.45 (1.08–1.95) 0.015	1.15 (0.81–1.63) 0.442
CKD	I	371	8.21	2.91 (2.47–3.43) <0.001	2.44 (2.06–2.91) <0.001	1.52 (1.22–1.91) <0.001
	II	278	5.98	2.11 (1.77–2.51) <0.001	1.59 (1.33–1.91) <0.001	1.12 (0.89–1.40) 0.351
	III	535	11.87	4.22 (3.62–4.93) <0.001	2.97 (2.53–3.50) <0.001	1.27 (1.00–1.60) 0.046
	IV	187	5.21	1.84 (1.51–2.23) <0.001	1.53 (1.26–1.85) <0.001	1.33 (1.05–1.69) 0.019
Death	I	502	10.33	2.23 (1.95–2.54) <0.001	1.49 (1.30–1.71) <0.001	1.15 (0.96–1.38) 0.135
	II	348	7.12	1.53 (1.32–1.77) <0.001	1.33 (1.15–1.55) <0.001	1.21 (1.00–1.47) 0.050
	III	662	13.37	2.90 (2.55–3.28) <0.001	1.76 (1.54–2.01) <0.001	1.19 (0.98–1.44) 0.082
	IV	267	7.13	1.53 (1.31–1.79) <0.001	1.29 (1.10–1.51) 0.001	1.24 (1.02–1.50) 0.034

Abbreviations: CHD, coronary heart disease; AF, atrial fibrillation; HF, heart failure; PAD, peripheral artery disease; CKD, chronic kidney disease; HR, hazard ratio; CI, confidence interval; PY, person-years. Notes: Hazard ratios (HRs) and 95% confidence intervals (CIs) were estimated from multivariable Cox proportional hazards models, with the non-MUO group serving as the reference. Model1: Crude unadjusted model. Model2: Adjusted for age, sex, ethnicity, smoking status, alcohol consumption. Model3: Further adjusted for systolic blood pressure, use of antihypertensive, glucose-lowering, and lipid-lowering medications, Townsend Deprivation Index, education level, and physical activity based on Model 2. Events are counts of incident events during follow-up. Rates are expressed per 1000 person-years of follow-up. *p*-values < 0.05 were considered statistically significant.

## Data Availability

The data that support the findings of this study are available from the UK Biobank but restrictions apply to the availability of these data, which were used under license for the current study, and so are not publicly available.

## Supplementary Materials

The following supporting information can be downloaded at: https://www.mdpi.com/article/10.3390/biomedicines14102358/s1, Figure S1: Flow chart of the selection of the participants from the UK Biobank; Figure S2: Model performance in training and validation sets; Figure S3: Indicators used to assess the number of clusters for PC1 and PC2 after PCA dimensionality reduction; Figure S4: Heatmap of metabolic profiles across subtypes; Figure S5: Principal component retention criteria for clustering dimensionality reduction; Method S1: Detailed Statistical Procedures for Feature Selection and Clustering; Method S2: Supplementary Statistical Analyses; Table S1: Variables and medication codes used to define metabolic syndrome in the UK Biobank cohort; Table S2: Detailed description of UK Biobank medication codes (mapped to ATC codes) used to define metabolic syndrome components; Table S3: Definitions and ICD-10/OPCS-4 codes for outcomes; Table S4: List of 64 NMR based metabolomic biomarkers in the UK Biobank; Table S5: Literature evidence supporting the relevance of 38 laboratory parameters to obesity or metabolic syndrome; Table S6: Sensitivity analysis of clustering stability across principal component retention strategies in the training set; Table S7: Validation-set consistency of the training-derived four-subtype solution; Table S8: Differential analysis of 67 metabolites across four metabolic subtypes (validation set); Table S9: Baseline characteristics of training-set and validation-set; Table S10: FDR corrected hazard ratio estimates for subtype outcome associations; Table S11: Associations of metabolic subtypes with CKM-related outcomes within MUO population; Table S12: Pairwise associations of metabolic subtypes with CKM-related outcomes within MUO population; Table S13: Fine Gray subdistribution hazard models for CKM related outcomes with all cause death as competing risk; Table S14: Subtype × Metabolic syndrome component interaction analyses for each clinical outcome; Table S15: Sex stratified hazard ratios for CHD and CKD by subtype, and sex by subtype interaction tests. References [51,52,53,54,55,56,57,58,59,60,61,62,63,64,65,66,67,68,69,70,71,72,73,74,75,76,77,78,79,80,81,82,83,84] are cited in the Supplementary Materials.

## Abbreviations

The following abbreviations are used in this manuscript:

AF	Atrial Fibrillation
ANOVA	Analysis of Variance
ApoA1	Apolipoprotein A1
ARI	Adjusted Rand Index
ATC	Anatomical Therapeutic Chemical
BCAA	Branched-Chain Amino Acid
BMI	Body Mass Index
CHD	Coronary Heart Disease
CKD	Chronic Kidney Disease
CKM	Cardiovascular–Kidney–Metabolic
CVD	Cardiovascular Disease
DHA	Docosahexaenoic Acid
eGFR	Estimated Glomerular Filtration Rate
FDR	False Discovery Rate
HF	Heart Failure
HbA1c	Glycated Hemoglobin
HDL(-C)	High-Density Lipoprotein (Cholesterol)
IQR	Interquartile Range
LA	Linoleic Acid
LASSO	Least Absolute Shrinkage and Selection Operator
LDL	Low-Density Lipoprotein
MetS	Metabolic Syndrome
MHO	Metabolically Healthy Obesity
MUO	Metabolically Unhealthy Obesity
NHS	National Health Service
NMI	Normalized Mutual Information
NMR	Nuclear Magnetic Resonance
PAD	Peripheral Artery Disease
PC	Principal Component
PCA	Principal Component Analysis
PUFA	Polyunsaturated Fatty Acid
TG	Triglyceride
VLDL	Very-Low-Density Lipoprotein
WC	Waist Circumference
