# Supplementary material for: Metabolically Unhealthy Obesity: Novel Subtypes Defined by Integrated Metabolomics and Hematology
Source: Biomedicines. 2026 Oct 16;14(10):2358. doi: 10.3390/biomedicines14102358 (PMC13701604; doi:10.3390/biomedicines14102358)
Supplement: Supplementary file 1 [file biomedicines-14-02358-s001.zip › biomedicines-4560096-supplementary.pdf]

Metabolically Unhealthy Obesity: Novel Subtypes Defined by Integrated Metabolomics and Hematology

Supplemental Material

Figure of contents:

- Figure S1: Flow chart of the selection of the participants from the UK Biobank.
- Figure S2: Model performance in training and validation sets.
- Figure S3: Indicators used to assess the number of clusters for PC1 and PC2 after PCA dimensionality reduction.
- Figure S4: Heatmap of metabolic profiles across subtypes.
- Figure S5: Principal component retention criteria for clustering dimensionality reduction.

Methods of contents:

- Supplemental Method S1: Detailed Statistical Procedures for Feature Selection and Clustering.
- Supplemental Method S2: Supplementary Statistical Analyses.

Table of contents:

- Supplemental Table S1: Variables and medication codes used to define metabolic syndrome in the UK Biobank cohort.
- Supplemental Table S2: Detailed description of UK Biobank medication codes (mapped to ATC codes) used to define metabolic syndrome components.
- Supplemental Table S3: Definitions and ICD-10/OPCS-4 codes for outcomes.
- Supplemental Table S4: List of 64 NMR-based metabolomic biomarkers in the UK Biobank.
- Supplemental Table S5: Literature evidence supporting the relevance of 38 laboratory parameters to obesity or metabolic syndrome.
- Supplemental Table S6: Sensitivity analysis of clustering stability across principal component retention strategies in the training set.
- Supplemental Table S7: Validation-set consistency of the training-derived four-subtype solution.
- Supplemental Table S8: Differential analysis of 67 metabolites across four metabolic subtypes (validation set).
- Supplemental Table S9: Baseline characteristics of training-set and validation-set.
- Supplemental Table S10: FDR corrected hazard ratio estimates for subtype outcome associations.
- Supplemental Table S11: Associations of metabolic subtypes with CKM-related outcomes within MUO population.
- Supplemental Table S12. Pairwise associations of metabolic subtypes with CKM-related outcomes within MUO population.
- Supplemental Table S13: Fine Gray subdistribution hazard models for CKM related outcomes with all cause death as competing risk.
- Supplemental Table S14: Subtype × Metabolic syndrome component interaction analyses for each clinical outcome.
- Supplemental Table S15: Sex stratified hazard ratios for CHD and CKD by subtype, and sex by subtype interaction tests.

Figure S1: Flow chart of the selection of the participants from the UK Biobank. Abbreviations: UKB, UK Biobank; BMI, body mass index.

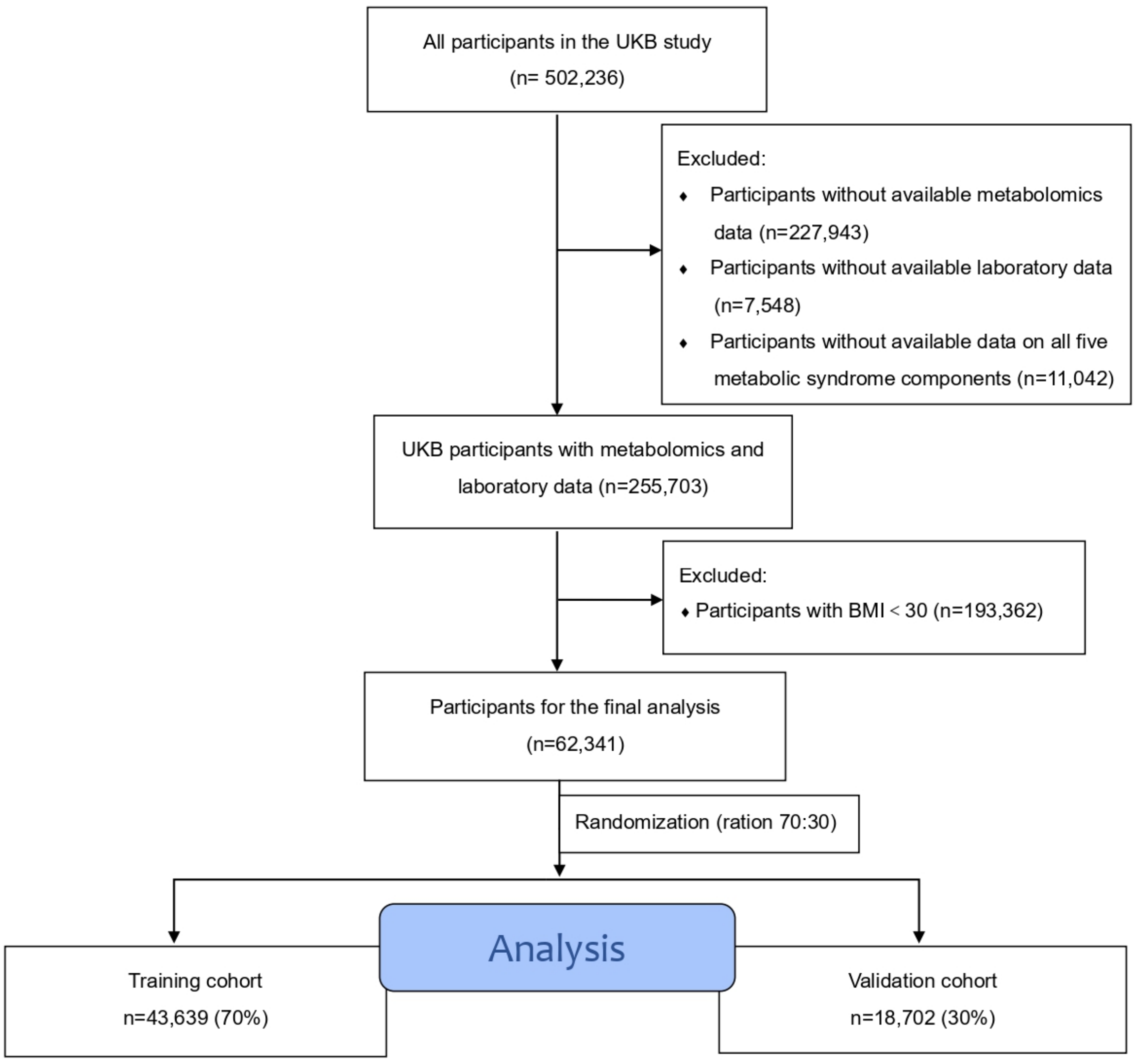

Figure S2: Model performance in training and validation sets. Notes: Receiver operating characteristic (ROC) curves demonstrating the performance of the 67-biomarker model in the training set (70%; AUC = 0.868, 95% CI 0.864–0.871; blue curve) and the validation set (30%; AUC = 0.866, 95% CI 0.860–0.871; red curve). The diagonal dashed line represents the reference line for random classification (AUC = 0.50). Abbreviations: AUC, area under the ROC curve; CI, confidence interval.

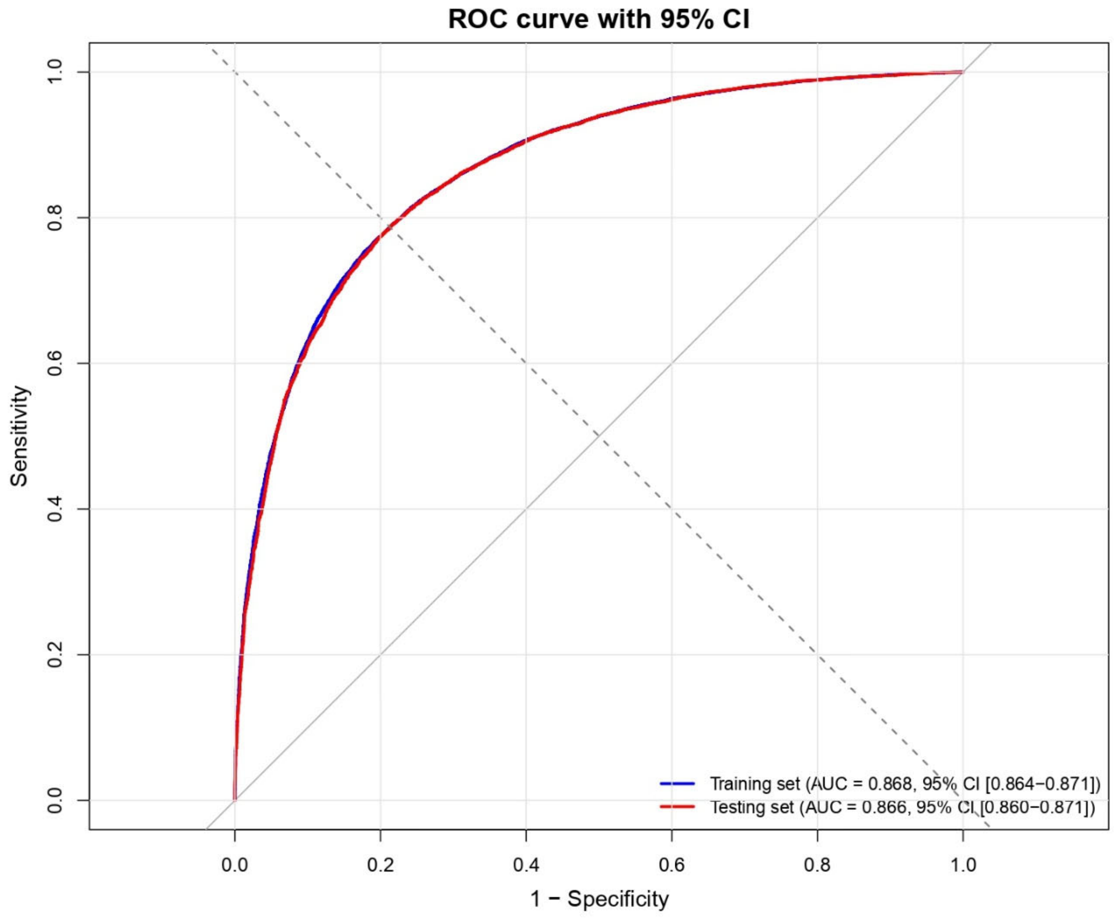

Figure S3: Indicators used to assess the number of clusters for PC1 and PC2 after PCA dimensionality reduction. A: Elbow chart; B: Silhouette Coefficient plot. Abbreviations: PCA, principal component analysis; PC, principal component; SSE, sum of squared errors.

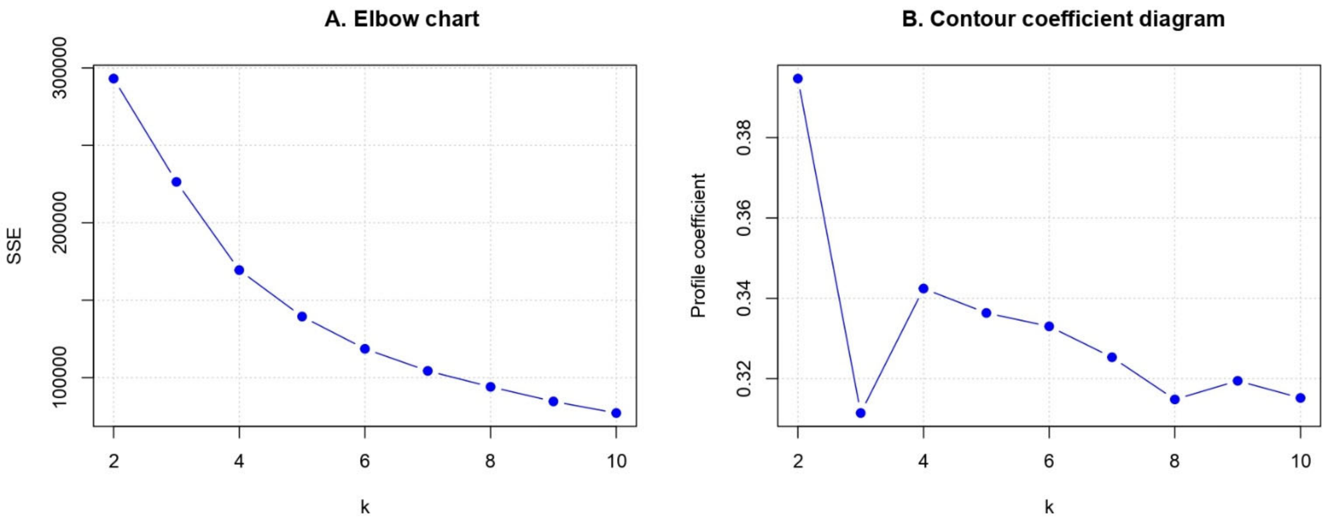

Figure S4: Heatmap of metabolic profiles across subtypes. Notes: Mean standardized levels (z-score) of 67 biomarkers across the four metabolic subtypes in the validation set (n = 13,044). Abbreviations of metabolite and biomarker names follow the list provided in Supplemental Table S8.

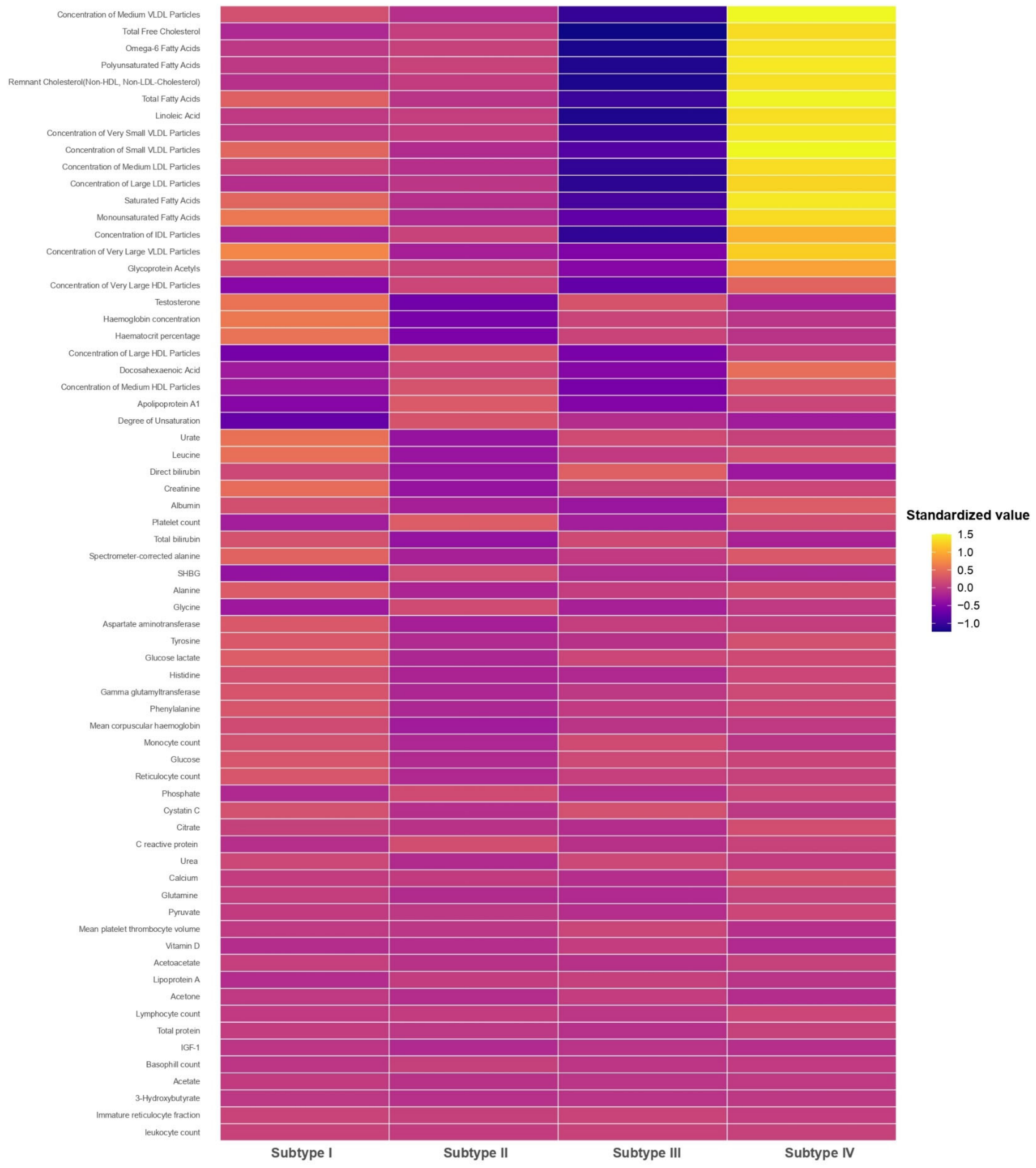

Figure S5: Principal component retention criteria for clustering dimensionality reduction. A: Scree plot; B: Cumulative variance plot. Notes: PCA was performed on the 67 LASSO-selected biomarkers in the training set (n = 30,533 MUO individuals). The scree plot (A) visualizes the marginal variance contribution of each successive PC, with the elbow point at PC2 indicating that subsequent components contribute progressively less unique information. The cumulative variance plot (B) quantifies the total information retained under different selection strategies. The Kaiser criterion (eigenvalue > 1) is a standard rule of thumb for retaining components that explain more variance than a single original variable. Abbreviations: PCA, principal component analysis; PC, principal component.

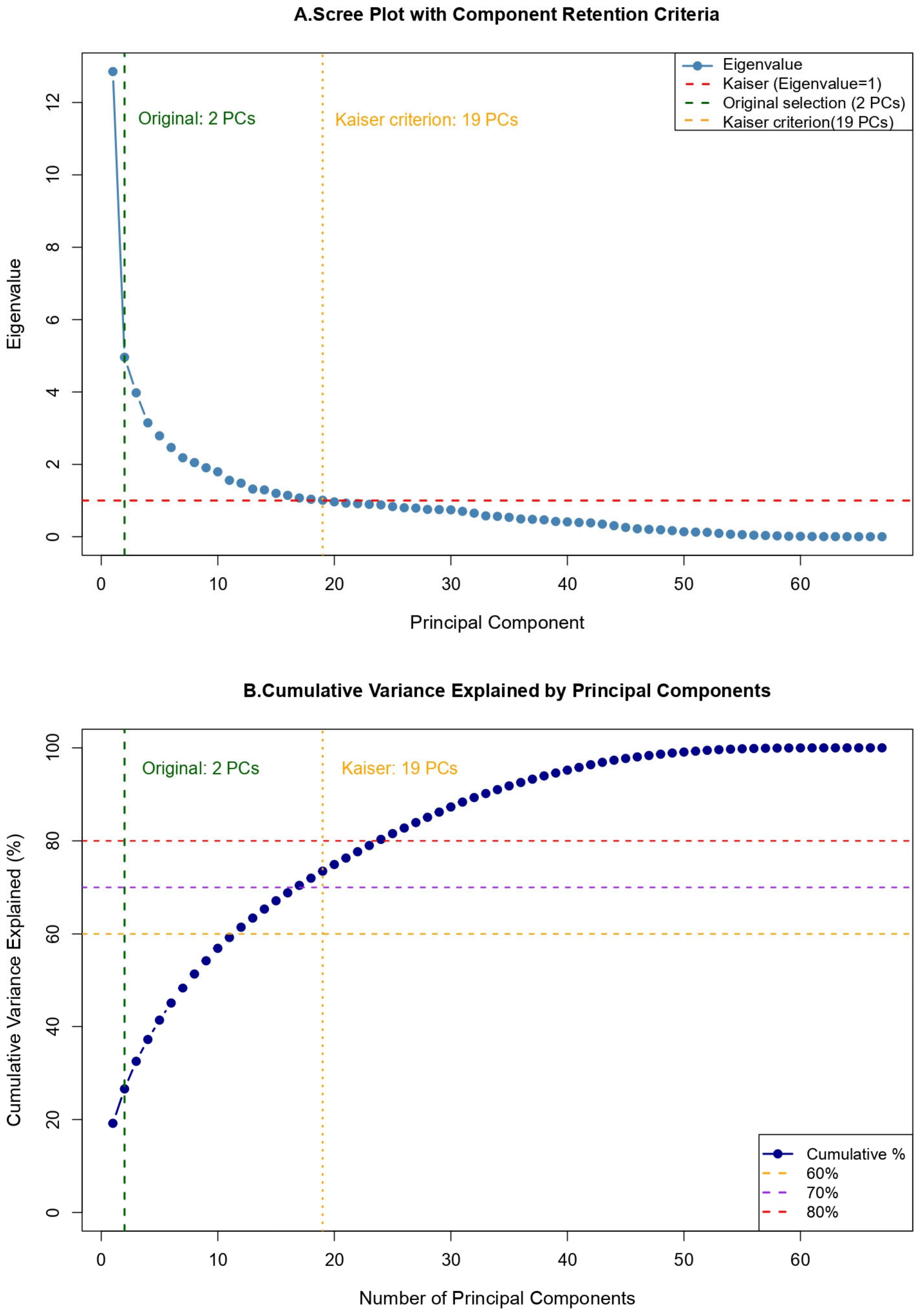

Supplemental Method S1: Detailed Statistical Procedures for Feature Selection and Clustering

Data partitioning and preprocessing. The analytical dataset (n=62,341) was randomly divided into training and testing sets using a 7:3 stratified split according to MUO status, yielding 43,639 and 18,702 participants, respectively. A total of 96 metabolic and hematological biomarkers were considered as candidate features. To reduce the influence of extreme biomarker values, continuous biomarkers were Winsorized at the 1st and 99th percentiles. The resulting variables were centered and scaled using means and standard deviations derived from the training set, which were subsequently applied unchanged to the testing set. These preprocessing procedures were performed before feature selection and clustering.

Supervised Feature Selection. To reduce redundancy among the 96 candidate biomarkers, LASSO logistic regression was first performed to discriminate MUO from non-MUO status in the training set. Five-fold cross-validation with the 1-standard-error (λ1se) criterion was used for feature selection, resulting in 67 biomarkers with non-zero coefficients. The selected feature set showed similar discrimination in the training and testing sets (AUC, 0.868 and 0.866, respectively). Because LASSO was optimized for MUO versus non-MUO discrimination rather than within-MUO heterogeneity, its potential influence on the clustering solution was examined in a sensitivity analysis using all 96 candidate biomarkers.

Dimensionality Reduction. PCA was performed on the 67 LASSO-selected biomarkers. The number of components to retain was evaluated using the scree plot, Kaiser criterion (eigenvalue >1), cumulative variance thresholds, and cluster separation across alternative numbers of principal components. PC1 and PC2 explained 19.19% and 7.40% of the total variance, respectively, accounting for 26.59% cumulatively. Although 19 components had eigenvalues >1, the primary analysis used PC1 and PC2 as a parsimonious representation of the dominant axes of metabolic variation. The scree plot showed an elbow at the second component (**Supplemental Figure S5**), and the 2-PC solution yielded the highest mean silhouette width (0.342) compared with higher-dimensional alternatives (**Supplemental Table S6**).

Clustering Algorithm. Clustering analyses were restricted to MUO participants in the training set (n=30,533). K-means clustering was applied to the PC1 and PC2 coordinates using Euclidean distance, with candidate solutions ranging from 2 to 10 clusters. The optimal number of clusters was evaluated using the within-cluster sum of squared errors (elbow method, **Supplemental Figure S3**) and mean silhouette width. The four-cluster solution was retained for the primary analysis, with a mean silhouette width of 0.342. The final K-means model used 25 random starts and a maximum of 100 iterations.

Sensitivity and Reproducibility Analyses. Several complementary analyses were conducted to assess the robustness of the clustering solution. First, to evaluate the potential influence of supervised feature selection, clustering based on all 96 candidate biomarkers was compared with the primary analysis based on 67 LASSO-selected biomarkers. Both approaches used PCA followed by four-cluster K-means clustering. The all-feature solution showed a similar mean silhouette width to the primary solution (0.346 vs. 0.342), with an ARI of 0.653. Because cluster labels are arbitrary, an optimal one-to-one label mapping was obtained using the Hungarian algorithm. Agreement was additionally assessed using a kappa coefficient and a label-matched cross-tabulation to facilitate direct assessment of subtype correspondence. The resulting kappa was 0.806. Following optimal label matching, the proportions of participants retaining the same subtype assignment were 82.5% for Subtype I (6,801/8,247), 76.5% for Subtype II (6,085/7,950), 93.7% for Subtype III (7,658/8,176), and 90.2% for Subtype IV (5,556/6,160). Second, to evaluate the influence of the number of retained principal components, clustering was repeated using 12, 17, and 19 PCs. The resulting solutions showed substantial agreement with the primary two-PC partition (ARI=0.848, 0.836, and 0.838, respectively; **Supplemental Table S6**). Direct K-means clustering in the standardized 67-dimensional biomarker space yielded an ARI of 0.838 and an NMI of 0.801 compared with the primary solution. Third, resampling-based reproducibility was assessed using 100 iterations, yielding mean ARI and NMI values of 0.961 and 0.944, respectively. These analyses were considered complementary assessments of clustering robustness rather than evidence of completely distinct biological classes.

Validation Set Assignment. Participants in the testing set were assigned to metabolic subtypes using centroid-based hard clustering. Each individual was projected onto the PC1–PC2 space derived from the training set and assigned to the nearest training-derived centroid based on Euclidean distance. The 2-PC solution showed high agreement in the independent testing set (ARI=0.936; **Supplemental Table S7**), supporting the reproducibility of the primary metabolic patterns across datasets.

Metabolic Signature Characterization. Standardized levels (z-scores) of the 67 selected biomarkers were compared across the four identified clusters using one-way analysis of variance. The Benjamini–Hochberg procedure was applied to control the FDR at P_{FDR}<0.05 (**Supplemental Table S8**).

Supplemental Method S2: Supplementary Statistical Analyses

Survival Analysis Specifications. For each outcome, the primary analysis was restricted to participants who were free of the corresponding disease at baseline. Specifically, participants with prevalent CHD, AF, HF, stroke, ischemic stroke, hemorrhagic stroke, PAD, or CKD were excluded from the respective outcome-specific risk set. Follow-up time was calculated from the baseline assessment date until the first occurrence of the outcome of interest, death, loss to follow-up, or the administrative censoring date, whichever occurred first. Cox proportional hazards models were fitted to estimate hazard ratios (HRs) and 95% confidence intervals (CIs) for each MUO subtype relative to the non-MUO reference group. Thus, the primary outcome analyses estimated associations with incident events. The proportional hazards assumption was formally assessed using Schoenfeld residuals; no violations were detected (all P>0.05). For the primary outcome analyses, the Benjamini–Hochberg procedure was applied across the 36 subtype–outcome comparisons to control the false discovery rate at P_{FDR}<0.05 (**Supplemental Table S10**). Additionally, within the MUO population, Cox proportional hazards models were fitted using Subtype IV as the reference group to directly compare the hazards of each other MUO subtype with Subtype IV (**Supplemental Table S11**). To directly evaluate whether outcome associations differed overall across the four MUO subtypes, global likelihood-ratio tests were performed within outcome-specific disease-free populations using the fully adjusted Model 3. Nominal evidence of heterogeneity was observed for atrial fibrillation

(P=0.029), heart failure (P=0.023), and chronic kidney disease (P=0.025). In addition, within the MUO population, multivariable Cox proportional hazards models were used to perform pairwise comparisons among all six subtype pairs (I vs II, I vs III, I vs IV, II vs III, II vs IV, and III vs IV) for each of the nine outcomes. These analyses used the same outcome-specific disease-free populations and covariate adjustment as the primary Model 3. Pairwise P-values were reported as nominal two-sided values without adjustment for multiple comparisons and were considered supplementary analyses for characterizing the direction and magnitude of subtype-specific differences (**Supplemental Table S12**).

Competing Risks Sensitivity Analyses. To account for competing events, Fine–Gray subdistribution hazard models were used for all outcomes except all-cause mortality, for which standard Cox proportional hazards models were applied because death was the endpoint of interest rather than a competing risk (**Supplemental Table S13**). For the remaining outcomes, death was treated as the competing event.

Interaction and Subgroup Analyses. Stratum-specific hazard ratios were estimated for each combination of MUO subtype and MetS component status. Likelihood ratio tests comparing nested Cox models with and without Subtype × MetS component interaction terms were used to formally assess potential effect modification, with adjustment for the same covariates as Model 3 (**Supplemental Table S14**). **S**ex-stratified analyses were additionally performed to examine potential effect modification by sex, with interaction P values derived from Subtype × Sex multiplicative interaction terms in the fully adjusted model (**Supplemental Table S15**).

Supplemental Table S1: Variables and medication codes used to define MetS in the UKB cohort.

**Metabolic syndrome component**	**Harmonized criteria (2009)**<br>***Three or more of the following:***	**UKB data field ID**	**Field code description**
**Elevated waist circumference**	Elevated waist circumference: ≥ 102 cm in males and ≥ 88 cm in females	48	Waist circumference
**Elevated triglycerides**	≥ 150 mg/dL (1.7 mmol/L)	30870	Triglycerides Blood Biochemistry;
**Elevated blood pressure**	Systolic: ≥130 mmHg and/or Diastolic ≥85 mmHg*, or antihypertensive medication use<br><br>*In UKB, BP was measured twice by trained nurses after participants had been at rest for at least 5 minutes. Individual systolic and diastolic BP measurements were averaged within a visit. Automated BP readings were the preferred source of data. However, if this was unavailable, manual readings were used	4080, 4079;<br><br>93,94	Systolic BP - automated reading, Diastolic BP - automated reading;<br><br>Systolic BP - manual reading, Diastolic BP - manual reading
		20003	ATC Codes starting with: C02, C03, C07, C08, C09
**Elevated blood glucose**	≥100 mg/dL (≥5.6 mmol/L) †, or drug treatment for elevated glucose level<br><br>†HbA1c used as a proxy indicator, with cut-offs based on the recommendations of the American Diabetes Association: HbA1c ≥5.7%	30750	Glycated haemoglobin (hbA1C)
		20003	ATC Codes starting with: A10
**Reduced HDL cholesterol**	Males: <40 mg/dL (1.0 mmol/L); Females: <50 mg/dL (1.3 mmol/L), or lipid-modifying medications †<br><br>†Since lipid-modifying medications can affect multiple lipid parameters, they were allocated exclusively to the reduced HDL-cholesterol component to avoid double counting	30760	HDL cholesterol
		20003	ATC Codes starting with: C10

Abbreviations: UKB = UK Biobank, ATC = Anatomical Therapeutic Chemical, HDL = High-density lipoprotein, BP = Blood pressure, HbA1c = Glycated haemoglobin.

Supplemental Table S2: Detailed description of UKB medication codes (mapped to ATC codes) used to define MetS components.

UKB Drug Category	UKB Drug Name	UKB Code	ATC Code
MEDICATIONS FOR REDUCED HDL CHOLESTEROL			
atorvastatin	Atorvastatin	1141146234	C10AA05
lipitor 10mg tablet	Atorvastatin	1141146138	C10AA05
rosuvastatin	Rosuvastatin	1141192410	C10AA07
crestor 10mg tablet	Rosuvastatin	1141192414	C10AA07
simvastatin	Simvastatin	1140861958	C10AA01
simvador 10mg tablet	Simvastatin	1141188146	C10AA01
zocor 10mg tablet	Simvastatin	1140881748	C10AA01
pravastatin	Pravastatin	1140888648	C10AA03

lipostat 10mg tablet	Pravastatin	1140861970	C10AA03
ezetimibe	Ezetimibe	1141192736	C10AX09
ezetrol 10mg tablet	Ezetimibe	1141192740	C10AX09
fenofibrate	Fenofibrate	1140861954	C10AB05
lipantil micro 67mg capsule	Fenofibrate	1141162544	C10AB05
supralip 160mg m/r tablet	Fenofibrate	1141172214	C10AB05
fluvastatin	Fluvastatin	1140888594	C10AA04
lescol 20mg capsule	Fluvastatin	1140864592	C10AA04
gemfibrozil	Gemfibrozil	1140861856	C10AB04
omacor 1g capsule	Omega-3-Acid Ethyl Esters	1141181868	C10AX06
bezafibrate	Bezafibrate	1140861924	C10AB02
questran 4g/sachet powder	Cholestyramine	1140861936	C10AC01
colestyramine	Cholestyramine	1140909780	C10AC01
ciprofibrate	Ciprofibrate	1140862026	C10AB08
bezalip-mono 400mg m/r tablet	Bezafibrate	1140861928	C10AB02
bezalip 200mg tablet	Bezafibrate	1140861926	C10AB02
niacin	Niacin	1140910670	C04AC01 |C10AD02
niaspan 500mg m/r tablet	Niacin	1141188546	C10AD02
nicotinic acid product	Niacin	1140861868	C04AC01 |C10AD02
cholestyramine	Cholestyramine	1140865576	C10AC01
fibrazate xl 400mg m/r tablet	Bezafibrate	1141201306	C10AB02
colestyramine product	Cholestyramine	1141180734	C10AC01
bezafibrate product	Bezafibrate	1141157260	C10AB02
modalim 100mg tablet	Ciprofibrate	1140862028	C10AB08
zimbacol xl 400mg m/r tablet	Bezafibrate	1141171548	C10AB02
colestipol	Colestipol	1140888590	C10AC02
colestid 5g/sachet granules	Colestipol	1140861848	C10AC02
acipimox	Acipimox	1140861892	C10AD06
colestyramine+aspartame 4g/sachet powder	Cholestyramine	1141180722	C10AC01
MEDICATIONS FOR ELEVATED BLOOD PRESSURE			
hydralazine	Hydralazine	1140888686	C02DB02
moxonidine	Moxonidine	1140928284	C02AC05
physiotens 200micrograms tablet	Moxonidine	1140928290	C02AC05
minoxidil	Minoxidil	1140860532	C02DC01 |D11AX01
doxazosin	Doxazosin	1140879778	C02CA04
methyldopa	Methyldopa	1140860470	C02AB
cardura 1mg tablet	Doxazosin	1140860690	C02CA04
doxadura 1mg tablet	Doxazosin	1141194372	C02CA04
clonidine	Clonidine	1140883468	C02AC01 |N02CX02 |S01EA04
indoramin	Indoramin	1140879782	C02CA02
prazosin	Prazosin	1140879794	C02CA01
hypovase 500mcg tablet	Prazosin	1140860580	C02CA01
indapamide	Indapamide	1140866078	C03BA11
natrilix sr 1.5mg m/r tablet	Indapamide	1141146378	C03BA11
hydrochlorothiazide	Hydrochlorothiazide	1140866162	C03AA03
furosemide	Furosemide	1140909708	C03CA01
frusemide	Furosemide	1140866116	C03CA01
spironolactone	Spironolactone	1140866236	C03DA01
spirolone 25mg tablet	Spironolactone	1140866318	C03DA01
chlortalidone	Chlorthalidone	1140909706	C03BA04
eplerenone	Eplerenone	1141201244	C03DA04
hygroton 50mg tablet	Chlorthalidone	1140866146	C03BA04
metolazone	Metolazone	1140866092	C03BA08
bendroflumethiazide	Bendroflumethiazide	1141194794	C03AA01
bendrofluazide	Bendroflumethiazide	1140866122	C03AA01
co-amilofruse	Amiloride |Furosemide	1140923402	C03EB01
bumetanide	Bumetanide	1140866280	C03CA02
co-amilozide	Amiloride |Hydrochlorothiazide	1140923276	C03EA01
amiloride	Amiloride	1140888512	C03DB01
bendroflumethiazide+potassium 2.5mg/7.7mmol m/r tablet	Bendroflumethiazide |Potassium	1141194800	C03AB01

frumil tablet	Amiloride |Furosemide	1140866406	C03CA01
bendrofluazide+potassium 2.5mg/7.7mmol m/r tablet	Bendroflumethiazide |Potassium	1140866450	C03AB01
bzt - bendrofluazide	Bendroflumethiazide	1140910442	C03AA01
dyazide tablet	Triamterene |Hydrochlorothiazide	1140866402	C03EA01
moduretic tablet	Amiloride |Hydrochlorothiazide	1140866420	C03EA01
navispare tablet	Amiloride |Cyclopenthiazide	1140866352	C03EA07
co-triamterzide	Triamterene |Hydrochlorothiazide	1140923272	C03EA01
torasemide	Torasemide	1140888496	C03CA04
moduret 25 tablet	Amiloride |Hydrochlorothiazide	1140866416	C03EA01
burinex a tablet	Bumetanide	1140866356	C03CA02
xipamide	Xipamide	1140866108	C03BA10
cyclopenthiazide	Cyclopenthiazide	1140866156	C03AA07
bisoprolol	Bisoprolol	1140879760	C07AB07
cardicor 1.25mg tablet	Bisoprolol	1141171152	C07AB07
atenolol	Atenolol	1140866738	C07AB03
tenormin 25 tablet	Atenolol	1140866756	C07AB03
metoprolol	Metoprolol	1140879818	C07AB02
carvedilol	Carvedilol	1140909368	C07AG02
propranolol	Propranolol	1140879842	C07AA05
timolol	Timolol	1140879866	C07AA06 |S01ED01
half-inderal la 80mg m/r capsule	Propranolol	1140866800	C07AA05
inderal 10mg tablet	Propranolol	1140866804	C07AA05
bedranol 10mg tablet	Propranolol	1140851556	C07AA05
half beta-prograne 80mg m/r capsule	Propranolol	1140866802	C07AA05
labetalol	Labetalol	1140879824	C07AG01
sotalol	Sotalol	1140879854	C07AA07
beta-cardone 40mg tablet	Sotalol	1140860304	C07AA07
co-tenidone	Chlorthalidone |Atenolol	1140923336	C07CB03
nebivolol	Nebivolol	1141164276	C07AB12
bisoprolol fumarate+hydrochlorothiazide 10mg/6.25mg tablet	Bisoprolol |Hydrochlorothiazide	1140864950	C07BB07
celiprolol	Celiprolol	1140879762	C07AB08
atenolol+bendroflumethiazide	Atenolol |Bendroflumethiazide	1141194810	C07BB03
nebilet 5mg tablet	Nebivolol	1141164280	C07AB12
propranolol hydrochloride+bendrofluazide 80mg/2.5mg capsule	Bendroflumethiazide |Propranolol	1140860418	C07BA05
sotalol hydrochloride+hydrochlorothiazide 80mg/12.5mg tablet	Sotalol |Hydrochlorothiazide	1140860332	C07BA07
tenoret 50 tablet	Chlorthalidone |Atenolol	1140860324	C07CB03
carteolol	Carteolol	1140879822	C07AA15 |S01ED05
betaxolol	Betaxolol	1140879758	C07AB05 |S01ED02
atenolol+bendrofluazide	Atenolol |Bendroflumethiazide	1141146126	C07BB03
tenif capsule	Atenolol |Nifedipine	1140860358	C07FB03
metoprolol tartrate+chlorthalidone 100mg/12.5mg tablet	Metoprolol |Chlorthalidone	1140860308	C07CB02
tenoretic tablet	Chlorthalidone |Atenolol	1140860328	C07CB03
beta-adalat capsule	Atenolol |Nifedipine	1140860356	C07FB03
celectol 200mg tablet	Celiprolol	1140860498	C07AB08
acebutolol	Acebutolol	1140866724	C07AB04
oxprenolol	Oxprenolol	1140879830	C07AA02
prindolol	Pindolol	1140910614	C07AA17
kalten capsule	Atenolol |Amiloride |Hydrochlorothiazide	1140860398	C07DB01
nadolol	Nadolol	1140860192	C07AA12
pindolol	Pindolol	1140860292	C07AA03
atenolol+chlortalidone	Chlorthalidone |Atenolol	1141180778	C07BB03
amlodipine	Amlodipine	1140879802	C08CA01
istin 5mg tablet	Amlodipine	1140861202	C08CA01
amlostin 5mg tablet	Amlodipine	1141200400	C08CA01
nifedipine	Nifedipine	1140861088	C08CA05

adalat 5mg capsule	Nifedipine	1140861090	C08CA05
coracten sr 10mg m/r capsule	Nifedipine	1140861120	C08CA05
adalate 10mg capsule	Nifedipine	1140881702	C08CA05
adipine mr 10 m/r tablet	Nifedipine	1140923572	C08CA05
fortipine la40 m/r tablet	Nifedipine	1141145870	C08CA05
nifedipress mr 10 m/r tablet	Nifedipine	1141157140	C08CA05
tensipine mr 10 m/r tablet	Nifedipine	1140927940	C08CA05
verapamil	Verapamil	1140888510	C08DA01
securon 40mg tablet	Verapamil	1140866466	C08DA01
half securon sr 120mg m/r tablet	Verapamil	1140866460	C08DA01
univer 120mg m/r capsule	Verapamil	1140881692	C08DA01
vertab sr 240 m/r tablet	Verapamil	1141169710	C08DA01
diltiazem	Diltiazem	1140879806	C05AE03 |C08DB01
tildiem 60mg m/r tablet	Diltiazem	1140861128	C08DB01
adizem-60 m/r tablet	Diltiazem	1140861138	C08DB01
adizem-xl plus m/r capsule	Diltiazem	1140926780	C08DB01
dilzem sr 60mg long acting m/r capsule	Diltiazem	1140861166	C08DB01
slozem 120mg m/r capsule	Diltiazem	1140911698	C08DB01
angitil sr 90 m/r capsule	Diltiazem	1140917428	C08DB01
viazem xl 120mg m/r capsule	Diltiazem	1141151474	C08DB01
zemtard 120 xl m/r capsule	Diltiazem	1141167832	C08DB01
calcicard 60mg tablet	Diltiazem	1140851730	C08DB01
felodipine	Felodipine	1140888646	C08CA02
cardioplen xl 5mg m/r tablet	Felodipine	1141199858	C08CA02
vascalpha 5mg m/r tablet	Felodipine	1141190160	C08CA02
felendil xl 5mg m/r tablet	Felodipine	1141188836	C08CA02
plendil 2.5mg m/r tablet	Felodipine	1140928212	C08CA02
felotens xl 5mg m/r tablet	Felodipine	1141188152	C08CA02
neofel xl 5mg m/r tablet	Felodipine	1141200782	C08CA02
felogen xl 5mg m/r tablet	Felodipine	1141188576	C08CA02
cabren 2.5mg m/r tablet	Felodipine	1141187094	C08CA02
lercanidipine	Lercanidipine	1141153026	C08CA13
zanidip 10mg tablet	Lercanidipine	1141153032	C08CA13
lacidipine	Lacidipine	1140861276	C08CA09
diltiazem hcl+hydrochlorothiazide 150mg/12.5mg m/r capsule	Diltiazem |Hydrochlorothiazide	1140926778	C08GA
nicardipine	Nicardipine	1140879810	C08CA04
motens 2mg tablet	Lacidipine	1140861282	C08CA09
cardene 20mg capsule	Nicardipine	1140861176	C08CA04
valsartan	Valsartan	1141145660	C09CA03
diovan 40mg capsule	Valsartan	1141145668	C09CA03
losartan	Losartan	1140916356	C09CA01
cozaar 25mg tablet	Losartan	1141179974	C09CA01
perindopril	Perindopril	1140888560	C09AA04
coversyl 2mg tablet	Perindopril	1140860802	C09AA04
lisinopril	Lisinopril	1140860696	C09AA03
zestril 2.5mg tablet	Lisinopril	1140860714	C09AA03
irbesartan	Irbesartan	1141152998	C09CA04
aprovel 75mg tablet	Irbesartan	1141153006	C09CA04
enalapril	Enalapril	1140888552	C09AA02
innovace 2.5mg tablet	Enalapril	1140860776	C09AA02
fosinopril	Fosinopril	1140888556	C09AA09
ramipril	Ramipril	1140860806	C09AA05
telmisartan	Telmisartan	1141166006	C09CA07
micardis 20mg tablet	Telmisartan	1141172492	C09CA07
tritace 1.25mg tablet	Ramipril	1141188408	C09AA05
lopace 2.5mg capsule	Ramipril	1141199940	C09AA05
candesartan cilexetil	Candesartan	1141156836	C09CA06
amias 2mg tablet	Candesartan	1141156846	C09CA06
cilazapril	Cilazapril	1140860882	C09AA08
olmesartan	Olmesartan	1141193282	C09CA08

losartan potassium+hydrochlorothiazide 50mg/12.5mg tablet	Losartan |Hydrochlorothiazide	1141151016	C09DA01
olmetec 10mg tablet	Olmesartan	1141193346	C09CA08
trandolapril	Trandolapril	1140860904	C09AA10
eprosartan	Eprosartan	1141171336	C09CA02
captopril	Captopril	1140860750	C09AA01
quinapril	Quinapril	1140860728	C09AA06
lisinopril+hydrochlorothiazide 10mg/12.5mg tablet	Lisinopril |Hydrochlorothiazide	1140864952	C09BA03
coaprovel 150mg/12.5mg tablet	Hydrochlorothiazide |Irbesartan	1141172686	C09DA04
enalapril maleate+hydrochlorothiazide 20mg/12.5mg tablet	Enalapril |Hydrochlorothiazide	1140860790	C09BA02
irbesartan+hydrochlorothiazide 150mg/12.5mg tablet	Hydrochlorothiazide |Irbesartan	1141172682	C09DA04
cozaar-comp 50mg/12.5mg tablet	Losartan |Hydrochlorothiazide	1141151018	C09DA01
perindopril+indapamide	Perindopril |Indapamide	1141180592	C09BA04
zestoretic 10 tablet	Lisinopril |Hydrochlorothiazide	1140864618	C09BA03
coversyl plus 4mg/1.25mg tablet	Perindopril |Indapamide	1141180598	C09BA04
co-diovan 80mg/12.5mg tablet	Valsartan |Hydrochlorothiazide	1141201040	C09DA03
valsartan+hydrochlorothiazide 80mg/12.5mg tablet	Valsartan |Hydrochlorothiazide	1141201038	C09DA03
teveten 300mg tablet	Eprosartan	1141171344	C09CA02
micardisplus 40mg/12.5mg tablet	Telmisartan |Hydrochlorothiazide	1141187790	C09DA07
telmisartan+hydrochlorothiazide 40mg/12.5mg tablet	Telmisartan |Hydrochlorothiazide	1141187788	C09DA07
felodipine+ramipril	Ramipril |Felodipine	1141165470	C09BB05
imidapril hydrochloride	Imidapril	1141164148	C09AA16
innozide tablet	Enalapril |Hydrochlorothiazide	1140860784	C09BA02
gopten 500micrograms capsule	Trandolapril	1140860912	C09AA10
capozide tablet	Hydrochlorothiazide |Captopril	1140881714	C09BA01
capoten 12.5mg tablet	Captopril	1140860758	C09AA01
carace 10 plus tablet	Lisinopril |Hydrochlorothiazide	1140864910	C09BA03
triapin mite 2.5mg/2.5mg tablet	Ramipril |Felodipine	1141165476	C09BB05
accupro 5mg tablet	Quinapril	1140881706	C09AA06
MEDICATIONS FOR ELEVATED BLOOD GLUCOSE			
metformin	Metformin	1140884600	A10BA02
glucophage 500mg tablet	Metformin	1140874686	A10BA02
insulin product	Insulin	1140883066	A10A
gliclazide	Gliclazide	1140874744	A10BB09
glyclizide	Gliclazide	1140910566	A10BB09
diamicron 80mg tablet	Gliclazide	1140874746	A10BB09
glimepiride	Glimepiride	1141152590	A10BB12
amaryl 1mg tablet	Glimepiride	1141156984	A10BB12
pioglitazone	Pioglitazone	1141171646	A10BG03
actos 15mg tablet	Pioglitazone	1141171652	A10BG03
glibenclamide	Glyburide	1140874718	A10BB01
repaglinide	Repaglinide	1141168660	A10BX02
rosiglitazone	Rosiglitazone	1141177600	A10BG02
rosiglitazone 1mg / metformin 500mg tablet	Metformin |Rosiglitazone	1141189090	A10BD03
avandamet 1mg / 500mg tablet	Metformin |Rosiglitazone	1141189094	A10BD03
glipizide	Glipizide	1140874646	A10BB07
avandia 4mg tablet	Rosiglitazone	1141177606	A10BG02
tolbutamide	Tolbutamide	1140874674	A10BB03 |V04CA01
acarbose	Acarbose	1140868902	A10BF01
nateglinide	Nateglinide	1141173882	A10BX03

Notes: Medication names, UKB codes, and corresponding ATC mappings are as catalogued by Qureshi et al. [32].

Supplemental Table S3: Definitions and ICD-10/OPCS-4 codes for outcomes.

	ICD-10	OPCS-4 Code
Coronary Atherosclerotic Heart Disease	I20, I21, I22, I23, I24, I25	K40–K46, K49, K50, K75
Atrial Fibrillation	I48	
Heart Failure	I50	
Stroke	I60, I61, I63, I64, I69	
Ischemic Stroke	I63	
Hemorrhagic Stroke	I60, I61	
Peripheral Artery Disease	I70, I73	
Chronic Kidney Disease	N18	

Abbreviations: ICD-10, International Statistical Classification of Diseases and Related Health Problems, version 10; OPCS-4, OPCS Classification of Interventions and Procedures, version 4.

Supplemental Table S4: List of 64 NMR-based metabolomic biomarkers in the UK Biobank.

Field ID	Metabolites	Unit	Group
23474	3-Hydroxybutyrate	mmol/L	Ketone bodies
23475	Acetate	mmol/L	Ketone bodies
23476	Acetoacetate	mmol/L	Ketone bodies
23477	Acetone	mmol/L	Ketone bodies
23460	Alanine	mmol/L	Amino acids
23479	Albumin	g/l	Fluid balance
23440	Apolipoprotein A1	g/l	Apolipoproteins
23439	Apolipoprotein B	g/l	Apolipoproteins
23473	Citrate	mmol/L	Glycolysis related metabolites
23404	Clinical LDL Cholesterol	mmol/L	Cholesterol
23481	Concentration of Chylomicrons and Extremely Large VLDL Particles	mmol/L	Lipoprotein subclasses
23430	Concentration of HDL Particles	mmol/L	Lipoprotein particle concentrations
23523	Concentration of IDL Particles	mmol/L	Lipoprotein subclasses
23429	Concentration of LDL Particles	mmol/L	Lipoprotein particle concentrations
23558	Concentration of Large HDL Particles	mmol/L	Lipoprotein subclasses
23530	Concentration of Large LDL Particles	mmol/L	Lipoprotein subclasses
23495	Concentration of Large VLDL Particles	mmol/L	Lipoprotein subclasses
23565	Concentration of Medium HDL Particles	mmol/L	Lipoprotein subclasses
23537	Concentration of Medium LDL Particles	mmol/L	Lipoprotein subclasses
23502	Concentration of Medium VLDL Particles	mmol/L	Lipoprotein subclasses
23572	Concentration of Small HDL Particles	mmol/L	Lipoprotein subclasses
23544	Concentration of Small LDL Particles	mmol/L	Lipoprotein subclasses
23509	Concentration of Small VLDL Particles	mmol/L	Lipoprotein subclasses
23429	Concentration of LDL Particles	mmol/L	Lipoprotein particle concentrations
23551	Concentration of Very Large HDL Particles	mmol/L	Lipoprotein subclasses
23488	Concentration of Very Large VLDL Particles	mmol/L	Lipoprotein subclasses
23516	Concentration of Very Small VLDL Particles	mmol/L	Lipoprotein subclasses
23478	Creatinine	mmol/L	Fluid balance
23443	Degree of Unsaturation	degree	Fatty acids
23450	Docosahexaenoic Acid	mmol/L	Fatty acids
23470	Glucose	mmol/L	Glycolysis related metabolites
20280	Glucose-lactate	mmol/L	Glycolysis related metabolites
23461	Glutamine	mmol/L	Amino acids
23462	Glycine	mmol/L	Amino acids
23480	Glycoprotein Acetyls	mmol/L	Inflammation
23463	Histidine	mmol/L	Amino acids
23465	Isoleucine	mmol/L	Amino acids
23405	LDL Cholesterol	mmol/L	Cholesterol
23471	Lactate	mmol/L	Glycolysis related metabolites
23466	Leucine	mmol/L	Amino acids
23449	Linoleic Acid	mmol/L	Fatty acids
23447	Monounsaturated Fatty Acids	mmol/L	Fatty acids

23444	Omega-3 Fatty Acids	mmol/L	Fatty acids
23445	Omega-6 Fatty Acids	mmol/L	Fatty acids
23468	Phenylalanine	mmol/L	Amino acids
23437	Phosphatidylcholines	mmol/L	Other lipids
23434	Phosphoglycerides	mmol/L	Other lipids
23446	Polyunsaturated Fatty Acids	mmol/L	Fatty acids
23472	Pyruvate	mmol/L	Glycolysis related metabolites
23402	Remnant Cholesterol(Non-HDL, Non-LDL-Cholesterol)	mmol/L	Cholesterol
23448	SaturatedFattyAcids	mmol/L	Fatty acids
20281	Spectrometer-corrected alanine	mmol/L	Amino acids
23438	Sphingomyelins	mmol/L	Other lipids
23400	Total Cholesterol	mmol/L	Cholesterol
23401	Total Cholesterol Minus HDL-C	mmol/L	Cholesterol
23436	TotalCholines	mmol/L	Other lipids
23464	Total Concentration of Branched-Chain Amino Acids(Leucine+Isoleucine+Valine)	mmol/L	Amino acids
23427	Total Concentration of Lipoprotein Particles	mmol/L	Lipoprotein particle concentrations
23415	Total Esterified Cholesterol	mmol/L	Cholesteryl esters
23442	Total Fatty Acids	mmol/L	Fatty acids
23419	Total Free Cholesterol	mmol/L	Free cholesterol
23469	Tyrosine	mmol/L	Amino acids
23403	VLDL Cholesterol	mmol/L	Cholesterol
23467	Valine	mmol/L	Amino acids

Supplemental Table S5: Literature evidence supporting the relevance of 38 laboratory parameters to obesity or metabolic syndrome.

Biomarker	Key Findings	Reference
Basophil count	Lower basophil count associated with higher odds of metabolic health obesity; OR 0.05 (95% CI 0.01–0.32) for Metabolically Healthy Obesity (MHO).	Li et al., 2025, Front. Nutr[1].
Haematocrit percentage	Haematocrit levels tend to increase with degree of obesity and blood pressure elevation.	Jeong et al., 2021, Sci. Rep[2].
Haemoglobin concentration	Higher haemoglobin associated with overweight/obesity (OR 1.4 and 1.3 respectively); positively correlated with Metabolic Syndrome (MetS) components.	Elmugabil et al., 2017, Future Sci. OA[3]
Immature reticulocyte fraction(IRF)	IRF positively associated with body mass index (BMI) and allometric body shape index (ABSI); obese-"apple" phenotype showed ~0.7 SD higher IRF compared to normal-weight-"pear" phenotype.	Christakoudi et al., 2023, BMC Endocr. Disord[4].
Lymphocyte count	Lower lymphocyte count associated with MHO; OR 0.77 (95% CI 0.67–0.89).	Li et al., 2025, Front. Nutr[1].
Mean corpuscular haemoglobin (MCH)	Higher MCH quartiles associated with lower ORs of obesity-related diseases.	Kohsari et al., 2021, Int. J. Endocrinol[5].
Mean platelet volume(MPV)	MPV significantly higher in obesity (MD 0.79 fL, 95% CI 0.42–1.16); associated with hypertriglyceridemia.	Nkambule et al., 2022, BMC Nutr[6].
Monocyte count	Absolute monocyte counts expanded in obesity; associated with BMI, body fat, waist circumference, triglycerides, and C-reactive protein.	Friedrich et al., 2019, Front. Immunol[7].
Neutrophil count	absolute neutrophil counts elevated in obesity; linear correlation with BMI; initiates inflammatory cascade in adipose tissue.	Gomez-Casado et al., 2025, Obes. Rev[8].
Platelet count	Platelet counts elevated in obese females; positive association with BMI and metabolic syndrome.	Samocha-Bonet et al., 2008, Mediators Inflamm[9].

Red blood cell (RBC) count	Higher RBC count quartiles associated with increased risk of obesity-related diseases.	Kohsari et al., 2021, Int. J. Endocrinol[5].
Reticulocyte count	Total reticulocyte count positively associated with BMI.	Christakoudi et al., 2023, BMC Endocr. Disord[4].
White blood cell (WBC) count	WBC count marker of subclinical inflammation; directly associated with both central adiposity and worsening glycemic status ($P < 0.05$).	Liu et al., 2024, Nutr. Diabetes[10]
Alanine aminotransferase (ALT)	ALT elevated in obesity; positively correlated with BMI, waist circumference, triglycerides, and fasting glucose.	Chen ZW et al., 2008, J. Zhejiang Univ. Sci. B[11]
Albumin	Higher serum albumin associated with increased risk of MetS and hypertension (OR 1.15 and 1.13 respectively) in Mendelian randomization studies.	Tang et al., 2025, Sci. Rep[12].
Alkaline phosphatase (ALP)	ALP levels higher in obese individuals; positively correlated with BMI ($r=0.627$, $p<0.01$); potential role in preadipocyte maturation.	Aqeel Rashid et al., 2021, Rep. Biochem. Mol. Biol[13].
Apolipoprotein A (ApoA)	Reduced ApoA-I levels associated with obesity; ApoA-I possesses anti-obesity effects via increased energy expenditure and UCP1 upregulation.	Ruan et al., 2011, J. Cell. Mol. Med[14].
Apolipoprotein B (ApoB)	ApoB gene restriction fragment length polymorphism haplotypes significantly associated with obesity ($p<0.005$).	Rajput-Williams et al., 1988, Lancet[15]
Aspartate aminotransferase (AST)	AST levels associated with obesity; less specific than ALT for fatty liver.	Jalili et al., 2022, Endocrinol. Diabetes Metab[16].
C-reactive protein (CRP)	CRP elevated in obesity; positively correlated with waist circumference, BMI, and metabolic syndrome; decreases with weight loss.	Cottam et al., 2004, Obes. Surg[17].
Calcium	Higher serum calcium associated with increased MetS risk in overweight/obese individuals (OR 1.63, 95% CI 1.06–2.53).	Chen JM et al., 2023, Arch. Endocrinol. Metab[18].
Cholesterol	Among morbidly obese women (BMI >40 kg/m^2), 39% had total serum cholesterol <5 mmol/L, indicating a significant proportion with normal levels.	Vierhapper et al., 2000, Metabolism[19].
Creatinine	Creatinine clearance positively correlated with BMI ($P < 0.001$).	Gerchman et al., 2009, J. Clin. Endocrinol. Metab[20].
Cystatin C	Cystatin C associated with obesity independent of gender; higher in type 2 diabetes with obesity.	Jhatta et al., 2020, Indian J. Endocrinol. Metab[21].
Direct bilirubin	Bilirubin has antioxidant properties; protects against adipocyte hypertrophy and improves endothelial function.	Žiberna et al., 2021, Antioxidants[22]
Gamma-glutamyl transferase (GGT)	GGT elevated in obesity; marker of oxidative stress and fatty liver; positively associated with BMI and metabolic syndrome.	Jalili et al., 2022, Endocrinol. Diabetes Metab[23].
Glucose	BMI dose-dependently associated with elevated fasting glucose and diabetes risk; overweight OR 1.67 (95% CI 1.36–2.06), obese OR 1.76 (95% CI 1.37–2.26); nonlinear J-shaped relationship ($p<0.001$).	Par et al., 2025, Health Sci. Rep[24].
Insulin-like Growth Factor 1(IGF-1)	Free IGF-1 increased in obesity despite normal total IGF-1; insulin-like growth factor binding protein 1 and insulin-like growth factor binding protein 2 suppressed; associated with insulin resistance.	Nam et al., 1997, Int. J. Obes[25].

Low Density Lipoprotein (LDL) direct	LDL mildly elevated in obesity; small dense LDL particles more atherogenic; obesity increases apoB and LDL particle concentration.	Bays et al., 2024, J Clin Lipidol [26]
Lipoprotein A(Lp(a))	Lp(a) polymorphisms associated with childhood obesity; elevated Lp(a) independent risk factor for cardiovascular disease.	Haksayar et al., 2025, Diagnostics[27]
Phosphate	Phosphate burden associated with chronic low-grade inflammation, vascular stiffness, and metabolic syndrome components.	Mironov et al., 2022, Nutrients[28]
Sex Hormone-Binding Globulin (SHBG)	SHBG independently predicts insulin resistance; OR 1.104 (95% CI 1.010–1.208) per unit decrease; inversely correlated with BMI (p=0.006).	Huang et al., 2023, Diabetes Ther[29].
Testosterone	Low testosterone associated with obesity and metabolic syndrome; bidirectional relationship; testosterone replacement reduces fat mass.	Liu L et al., 2021, Diabetes Metab. Syndr. Obes[30].
Total bilirubin	Higher total bilirubin associated with reduced MetS risk; low bilirubin predicts MetS; modifies obesity-stroke relationship.	El-Eshmawy et al., 2024, Porto Biomed. J[31].
Urate	Strong connection between obesity and hyperuricemia; associated with metabolic syndrome.	Timsans et al., 2024, J. Clin. Med[32].
Total protein	Total protein significantly associated with insulin resistance risk across all subgroups; association particularly pronounced in participants with BMI ≥25 kg/m².	Zeng et al., 2024, Front. Endocrinol[33].
Urea	One standard deviation (SD ≈ 4.8 kg/m2) genetically higher BMI was associated with increased blood urea nitrogen.	Kjaergaard et al., 2022, Clin. Chem[34].
Vitamin D	Vitamin D deficiency prevalent in obesity (pooled OR 3.43, 95% CI 2.33–5.06); volumetric dilution and sequestration in adipose tissue.	Vanlint et al., 2013, Nutrients[35].

Supplemental Table S6: Sensitivity analysis of clustering stability across principal component retention strategies in the training set.

Strategy	PCs Retained	Cumulative Variance (%)	Silhouette Width	ARI vs PC2	ARI vs 67D
PC2	2	26.6	0.343	1.000	0.838
PC_60pct	12	61.4	0.112	0.848	0.969
PC_70pct	17	70.4	0.095	0.836	0.976
PC_Kaiser	19	73.5	0.091	0.838	0.980

Abbreviations: PC, principal component; ARI, adjusted Rand index.

Notes: ARI vs PC2 indicates agreement with the original 2-PC clustering solution. ARI vs 67D indicates agreement with direct clustering on the full 67 standardized biomarkers.

Supplemental Table S7: Validation-set consistency of the training-derived four-subtype solution.

Comparison	ARI	Interpretation
Testing-set 2PC vs Training-set 2PC projection	0.936	Cross-dataset reproducibility of 2-PC clustering
Testing-set 67D vs Training-set 67D projection	0.937	Cross-dataset reproducibility of 67D clustering
Testing-set 2PC vs Testing-set 67D	0.864	Testing-set consistency across dimensionalities
Training-set 2PC vs Training-set 67D	0.838	Training-set consistency across dimensionalities

Abbreviations: ARI, adjusted Rand Index; PC, principal component; 67D, 67-dimensional standardized biomarker matrix.

Notes: All ARI values > 0.80 indicate strong agreement.

Supplemental Table S8: Differential analysis of 67 metabolites across four metabolic subtypes (validation set).

Metabolite Name	Subtype I Mean±SD	Subtype II Mean±SD	Subtype III Mean±SD	Subtype IV Mean±SD	ANOVA F value	ANOVA P value	FDR adjusted P value
Linoleic Acid	**-1.0692±0.4915**	-0.0017±0.5771	0.07±0.5432	1.3267±0.728	8562.95	< 1.00E-300	< 1.00E-300
Omega 6 Fatty Acids	**-1.1056±0.483**	0.0421±0.5365	0.044±0.5078	1.3529±0.6898	10053.6	< 1.00E-300	< 1.00E-300
Concentration of Small VLDL Particles	**-0.9132±0.5201**	-0.3829±0.6255	0.3357±0.5568	1.259±0.8045	6877.21	< 1.00E-300	< 1.00E-300
Polyunsaturated Fatty Acids	**-1.097±0.4909**	0.0357±0.5367	0.0434±0.5108	1.3505±0.7025	9751.83	< 1.00E-300	< 1.00E-300
Concentration of Large LDL Particles	**-0.996±0.4931**	-0.0335±0.6281	0.0197±0.5874	1.3381±0.7459	7340.01	< 1.00E-300	< 1.00E-300
Concentration of Medium VLDL Particles	**-1.0185±0.4695**	-0.2413±0.6059	0.2187±0.4963	1.3712±0.6828	9493.68	< 1.00E-300	< 1.00E-300
Remnant Cholesterol	**-1.0674±0.4544**	0.0021±0.6032	0.0247±0.5289	1.3799±0.651	9677.72	< 1.00E-300	< 1.00E-300
Concentration of Medium LDL Particles	**-0.9914±0.5011**	-0.1411±0.6167	0.1379±0.6086	1.3135±0.7472	7165.05	< 1.00E-300	< 1.00E-300
Concentration of Very Large VLDL Particles	**-0.6645±0.6263**	-0.5626±0.6153	0.5443±0.7641	0.8832±0.9982	3406.62	< 1.00E-300	< 1.00E-300
Concentration of Large HDL Particles	**-0.474±0.756**	0.6605±1.1069	-0.4649±0.5743	0.3937±0.9303	1565.02	< 1.00E-300	< 1.00E-300
Concentration of Small LDL Particles	**-1.0066±0.49**	-0.1884±0.6233	0.1772±0.545	1.3424±0.7117	8193.57	< 1.00E-300	< 1.00E-300
HDL Cholesterol	**-0.5569±0.7736**	0.6293±1.0081	-0.4162±0.6615	0.4791±0.9372	1695.27	< 1.00E-300	< 1.00E-300
Total Triglycerides	**-0.6845±0.6203**	-0.5241±0.5929	0.5213±0.7875	0.8906±1.0154	3301.45	< 1.00E-300	< 1.00E-300
Sphingomyelins	**-1.0359±0.5625**	0.3053±0.5995	-0.1917±0.5539	1.2343±0.7398	7394.69	< 1.00E-300	< 1.00E-300
Testosterone	**0.3283±0.9784**	-0.5903±0.7347	0.4575±0.9331	-0.2816±0.9345	1040.92	< 1.00E-300	< 1.00E-300
Concentration of Very Large HDL Particles	**-0.6543±0.7371**	0.3847±1.0264	-0.2647±0.6833	0.7229±0.9347	1678.41	< 1.00E-300	< 1.00E-300
Apolipoprotein A	**-0.4149±0.8276**	0.548±1.0128	-0.3839±0.8013	0.3528±0.9616	1021.09	< 1.00E-300	< 1.00E-300
Phosphatidylcholines	**-1.0131±0.5796**	0.1745±0.6937	-0.0358±0.6032	1.1652±0.8022	5487.57	< 1.00E-300	< 1.00E-300
LDL Cholesterol	**-1.0301±0.5361**	0.024±0.5897	0.0092±0.5848	1.3228±0.7157	7665.77	< 1.00E-300	< 1.00E-300
Degree of Unsaturation	**-0.0129±0.9983**	0.5437±0.8732	-0.5208±0.8981	0.0084±0.9088	774.076	< 1.00E-300	< 1.00E-300
Monounsaturated Fatty Acids	**-0.816±0.6176**	-0.3809±0.5523	0.4298±0.7923	1.0017±0.9475	3847.49	< 1.00E-300	< 1.00E-300
Leucine	**-0.0373±0.933**	-0.487±0.7498	0.4628±1.0437	0.0624±1.0107	601.045	< 1.00E-300	< 1.00E-300
Haematocrit percentage	**0.1455±1.0266**	-0.4828±0.8682	0.4112±0.9372	-0.1166±0.9172	567.547	< 1.00E-300	< 1.00E-300
Docosahexaenoic Acid	**-0.4641±0.7489**	0.2242±0.8988	-0.2041±0.8999	0.5976±1.1503	795.525	< 1.00E-300	< 1.00E-300
Glycoprotein Acetyls	**-0.5795±0.8964**	-0.1514±0.8387	0.1982±0.8885	0.7001±0.9538	1128.67	< 1.00E-300	< 1.00E-300
Concentration of IDL Particles	**-0.9415±0.5463**	0.124±0.7258	-0.0671±0.6983	1.1777±0.8003	4773.51	< 1.00E-300	< 1.00E-300
Urea	**0.0853±1.1237**	-0.1657±0.823	0.1069±1.1085	-0.0412±0.8384	55.5415	1.14E-35	1.53E-35
Immature reticulocyte fraction	**0.0414±1.0256**	-0.0663±0.9973	0.0705±0.9938	-0.0631±0.9681	16.452	1.15E-10	1.31E-10
Lipoprotein A	**0.0553±1.0447**	0.0925±1.0659	-0.1151±0.9027	-0.0394±0.9573	30.5401	1.15E-19	1.43E-19
Calcium	**-0.1559±1.0031**	-0.0006±0.9843	0.0051±0.9927	0.2008±0.99	64.9058	1.19E-41	1.62E-41

Histidine	**-0.1255±0.952**	-0.2215±0.9015	0.2206±1.0915	0.1588±0.9683	159.656	1.21E-101	2.00E-101	
Glycine	**-0.1868±0.8741**	0.2511±1.1566	-0.165±0.8173	0.1428±1.0655	169.182	1.30E-107	2.31E-107	
Phosphate	**-0.1157±1.0042**	0.16±0.9148	-0.1248±1.0609	0.1129±0.9742	75.8013	1.32E-48	1.84E-48	
Acetoacetate	**-0.0775±1.0163**	0.0023±1.1131	0.0256±0.9238	0.0654±0.9113	11.6015	1.37E-07	1.52E-07	
Glucose lactate	**0.0346±1.0978**	-0.261±0.5568	0.2279±1.2851	-0.012±0.7702	145.228	1.40E-92	2.16E-92	
Total protein	**-0.0603±1.0342**	-0.0147±0.988	0.017±0.9954	0.0763±0.9698	10.0015	1.40E-06	1.53E-06	
Urate	**0.1028±1.0096**	-0.4396±0.8452	0.3653±0.9763	-0.0545±0.9809	431.848	1.44E-267	3.66E-267	
Creatinine	**0.0204±1.0015**	-0.3862±0.5412	0.3484±1.3562	0.008±0.6601	338.195	1.52E-211	3.49E-211	
Aspartate aminotransferase	**0.0282±1.0289**	-0.2695±0.6731	0.2331±1.1639	0.0004±0.9952	153.116	1.54E-97	2.49E-97	
Vitamin D	**0.1135±1.09**	0.0289±1.0013	-0.0974±0.9765	-0.0578±0.8811	30.3343	1.56E-19	1.90E-19	
SHBG	**-0.0539±0.8717**	0.3646±1.2055	-0.2992±0.7363	-0.0013±1.0239	277.303	1.59E-174	3.22E-174	
IGF 1	**-0.0044±1.0461**	-0.0364±0.9828	0.037±0.9959	0.0036±0.963	3.17	2.33E-02	2.43E-02	
HbA1c	**0.239±1.1622**	-0.1978±0.629	0.1022±1.1996	-0.1971±0.7438	163.975	2.38E-104	4.02E-104	
Gamma glutamyltransferase	**-0.0437±0.9421**	-0.2118±0.6898	0.1756±1.1377	0.0978±1.1511	100.545	2.41E-64	3.48E-64	
Phenylalanine	**-0.0077±0.9943**	-0.286±0.8702	0.2656±1.0496	0.0259±0.997	184.75	2.43E-117	4.43E-117	
Monocyte count	**0.1369±0.9691**	-0.1854±0.7243	0.1569±1.3102	-0.1508±0.7845	113.112	2.60E-72	3.92E-72	
Pyruvate	**-0.1279±0.9651**	-0.0025±1.0196	0.0444±0.9977	0.1135±1.0049	33.0598	2.80E-21	3.55E-21	
Direct bilirubin	**0.4107±1.3159**	-0.2575±0.7004	0.0649±0.9397	-0.2977±0.6662	387.658	2.87E-241	6.79E-241	
Red blood cell erythrocyte count	**0.1335±1.0362**	-0.4133±0.8658	0.3608±0.9739	-0.1234±0.9287	419.478	3.14E-260	7.68E-260	
Isoleucine	**-0.0571±0.9231**	-0.4331±0.7544	0.417±1.0747	0.0799±1.0307	475.359	3.15E-293	8.28E-293	
High light scatter reticulocyte count	**0.0663±1.0166**	-0.2578±0.8609	0.2267±1.0963	-0.0567±0.9266	149.419	3.24E-95	5.11E-95	
Alanine aminotransferase	**-0.0131±0.9483**	-0.3724±0.6451	0.3494±1.172	0.0332±1.0238	326.062	3.32E-204	7.37E-204	
Basophill count	**-0.0162±0.874**	0.042±1.0694	-0.0206±1.1439	-0.0054±0.8441	2.8572	3.56E-02	3.66E-02	
Lymphocyte count	**-0.0368±0.6108**	-0.0054±0.5266	0.0237±1.6787	0.0242±0.5799	2.7906	3.90E-02	3.95E-02	
C reactive protein	**-0.0675±1.0258**	0.1096±1.1353	-0.0641±0.905	0.0331±0.8782	25.0044	4.00E-16	4.81E-16	
Neutrophill count	**0.074±1.0272**	-0.0332±1.0121	0.0197±0.9864	-0.0815±0.9574	14.0185	4.03E-09	4.54E-09	
Platelet count	**-0.2653±0.9414**	0.2779±1.0257	-0.1588±0.9235	0.2038±1.0061	250.499	4.44E-158	8.76E-158	
Acetate	**-0.0089±0.7888**	-0.0162±0.9551	0.0191±1.0977	0.0071±1.1581	0.8658	4.58E-01	4.58E-01	
X3 Hydroxybutyrate	**-0.0441±0.9757**	0.0338±1.1587	-0.0226±0.8621	0.0449±0.9784	5.9622	4.67E-04	4.95E-04	
Glucose	**0.0619±1.0899**	-0.2334±0.5767	0.1893±1.2906	-0.0324±0.7766	112.68	4.87E-72	7.21E-72	
Cystatin C	**0.1423±1.1576**	-0.1631±0.7226	0.1217±1.1819	-0.1399±0.7338	90.6121	4.91E-58	6.98E-58	
Mean platelet thrombocyte volume	**0.1102±1.0302**	-0.0212±0.9867	-0.0172±0.9858	-0.0956±0.9827	23.2344	5.41E-15	6.40E-15	
Tyrosine	**-0.0575±1.0002**	-0.258±0.8976	0.254±1.0226	0.0712±1.0047	166.541	5.86E-106	1.02E-105	
Acetone	**0.0567±0.8847**	0.0007±1.36	-0.0095±0.7987	-0.0633±0.8144	7.4113	5.88E-05	6.33E-05	
Albumin	**-0.294±0.9986**	-0.165±0.9241	0.1665±0.9302	0.3813±1.0207	314.065	6.25E-197	1.34E-196	
Total bilirubin	**0.2295±1.1426**	-0.2763±0.7725	0.1677±1.0535	-0.1704±0.8581	217.864	6.27E-138	1.20E-137	
Spectrometer corrected alanine	**-0.0637±1.0608**	-0.3539±0.8613	0.3131±1.0077	0.1248±0.9136	295.801	8.05E-186	1.68E-185	
Alanine	**-0.0045±1.0707**	-0.2979±0.8864	0.2561±1.0052	0.0499±0.9303	188.715	8.19E-120	1.53E-119	
Mean corpuscular haemoglobin	**0.0049±1.0611**	-0.1431±1.0616	0.1159±0.9191	0.024±0.912	40.138	8.23E-26	1.06E-25	
Glutamine	**-0.0916±1.0351**	-0.0861±0.9493	0.0699±0.9992	0.1396±0.9949	41.6116	9.38E-27	1.23E-26	
Alkaline phosphatase	**-0.0651±1.0114**	0.0864±1.0356	-0.0631±0.9369	0.0586±1.0079	21.292	9.41E-14	1.10E-13	

Abbreviations: VLDL, Very-Low-Density Lipoprotein; LDL, Low-Density Lipoprotein; HDL, High-Density Lipoprotein; IDL: Intermediate-Density Lipoprotein; SHBG, Sex Hormone-Binding Globulin; IGF-1: Insulin-like Growth Factor 1.

Notes: Data are presented as mean ± SD of standardized (z-score normalized) values for each metabolite within each subtype. Standardization was performed by subtracting the mean and dividing by the standard deviation across all MUO individuals in the validation set. Of the 67 biomarkers examined, 66 differed significantly across subtypes after FDR correction ($P_{FDR} < 0.05$). Leukocyte count was the sole non-significant metabolite ($P_{FDR} = 0.057$).

Supplemental Table S9: Baseline Characteristics of training-set and validation-set.

Variable	Training-set (N = 43,639)	Validation-set (N = 18,702)	P-value	SMD
Age (years)(mean (SD))	56.88 (7.88)	56.84 (7.85)	0.609	0.004
Sex(%)				
Female	22686 (52.0%)	9702 (51.9%)	0.810	0.002
Male	20953 (48.0%)	9000 (48.1%)	0.810	0.002
SBP(mmHg) (mean (SD))	143.79 (18.88)	143.71 (18.60)	0.632	0.004
DBP(mmHg) (mean (SD))	85.83 (10.35)	85.94 (10.34)	0.238	0.011
WC(cm) (mean (SD))	104.96 (11.10)	104.92 (11.01)	0.704	0.003
BMI (kg/m²)(median [IQR])	33.92 (3.87)	33.96 (3.88)	0.275	0.010
Smoking status (%)				
Never	22086 (50.9%)	9464 (50.9%)	0.973	0.002
Former	17107 (39.4%)	7325 (39.4%)	0.973	0.002
Current	4178 (9.6%)	1802 (9.7%)	0.973	0.002
Alcohol drinker status (%)				
Never	2351 (5.4%)	1042 (5.6%)	0.651	0.008
Former	1987 (4.6%)	853 (4.6%)	0.651	0.008
Current	39168 (90.0%)	16754 (89.8%)	0.651	0.008
Ethnic (%)				
White	38686 (88.7%)	16607 (88.8%)	0.746	0.007
Non White	4901 (11.2%)	2076 (11.1%)	0.746	0.007
TG(mmol/L)(median [IQR])	2.15 (1.17)	2.13 (1.15)	0.159	0.012
HDL-C(mmol/L)(median [IQR])	1.28 (0.31)	1.28 (0.31)	0.627	0.004
HbA1c(mmol/mol)(median [IQR])	38.51 (8.89)	38.50 (8.88)	0.908	0.001

Abbreviations: DBP, diastolic blood pressure; SBP, systolic blood pressure; WC, waist circumference; BMI, body mass index; HDL-C, high-density lipoprotein cholesterol; TG, triglycerides; HbA1c, glycated hemoglobin; SMD, standardized mean difference.

Notes: Means (SD) are reported for continuous variables; in contrast, count (%) is presented for categorical features. Continuous variables were compared using the Kruskal–Wallis rank-sum test, and categorical variables were analyzed with Pearson's chi-squared test. P-values and standardized mean difference (SMD) were used to determine the statistical significance of differences between the training set and validation set.

Supplemental Table S10: FDR-corrected hazard ratio estimates for subtype-outcome associations.

Outcome	Subtype	HR (95% CI)	P-value	P_{FDR}
CHD	I	1.15 (0.97-1.37)	0.104	0.288
	II	1.34 (1.11-1.61)	0.002	**0.024**
	III	1.21 (1.01-1.46)	0.042	0.180
	IV	1.37 (1.15-1.64)	0.001	**0.011**
AF	I	0.91 (0.76-1.08)	0.275	0.550
	II	1.00 (0.83-1.20)	0.962	0.962
	III	0.97 (0.80-1.16)	0.723	0.828
	IV	0.73 (0.60-0.90)	0.003	**0.027**
HF	I	1.08 (0.84-1.38)	0.557	0.743
	II	1.13 (0.87-1.47)	0.375	0.586
	III	1.42 (1.10-1.82)	0.006	**0.046**
	IV	1.08 (0.82-1.42)	0.590	0.755
Stroke	I	1.17 (0.85-1.60)	0.344	0.574
	II	1.17 (0.84-1.63)	0.344	0.574
	III	1.06 (0.75-1.50)	0.736	0.828
	IV	0.89 (0.62-1.28)	0.519	0.719
Ischemic Stroke	I	1.10 (0.77-1.57)	0.608	0.755
	II	1.33 (0.92-1.93)	0.128	0.325
	III	1.01 (0.68-1.50)	0.953	0.962
	IV	0.91 (0.61-1.37)	0.663	0.796
Hemorrhagic Stroke	I	1.43 (0.73-2.81)	0.299	0.566
	II	0.59 (0.27-1.28)	0.178	0.401
	III	1.10 (0.53-2.27)	0.796	0.868
	IV	0.59 (0.25-1.40)	0.231	0.489
PAD	I	1.01 (0.72-1.42)	0.951	0.962
	II	0.70 (0.48-1.03)	0.069	0.227

	III	0.86 (0.60-1.23)	0.396	0.594
	IV	1.15 (0.81-1.63)	0.442	0.636
**CKD**	I	1.52 (1.22-1.91)	0.000	**0.009**
	II	1.12 (0.89-1.40)	0.351	0.574
	III	1.27 (1.00-1.60)	0.046	0.180
	IV	1.33 (1.05-1.69)	0.019	0.117
**Death**	I	1.15 (0.96-1.38)	0.135	0.325
	II	1.21 (1.00-1.47)	0.050	0.180
	III	1.19 (0.98-1.44)	0.082	0.247
	IV	1.24 (1.02-1.50)	0.034	0.174

Abbreviations: CHD, coronary heart disease; AF, atrial fibrillation; HF, heart failure; PAD, peripheral artery disease; CKD, chronic kidney disease; HR, hazard ratio; CI, confidence interval; FDR, false discovery rate; P_{FDR}, Benjamini-Hochberg adjusted P-value.

Notes: Benjamini–Hochberg correction was applied across the 36 prespecified subtype–outcome comparisons. All models were adjusted for age, sex, ethnicity, smoking status, alcohol consumption, systolic blood pressure, use of antihypertensive, glucose-lowering, and lipid-lowering medications, Townsend Deprivation Index, education level, and physical activity. Non-MUO served as the reference group (HR = 1.00 for all outcomes). P-values < 0.05 were considered statistically significant. P_{FDR} indicate statistical significance after FDR correction (P_{FDR} < 0.05).

Supplemental Table S11. Associations of metabolic subtypes with CKM-related outcomes within MUO population.

Outcome	Subtype	HR (95% CI)	P-value	P_{FDR}
**CHD**	I	0.86 (0.71-1.04)	0.117	0.395
	II	0.96 (0.79-1.16)	0.662	0.850
	III	0.91 (0.74-1.10)	0.324	0.583
	IV	Reference	-	-
**AF**	I	1.24 (1.00-1.54)	0.054	0.292
	II	1.36 (1.09-1.70)	0.006	0.167
	III	1.33 (1.06-1.67)	0.013	0.177
	IV	Reference	-	-
**HF**	I	1.03 (0.78-1.36)	0.845	0.929
	II	1.02 (0.77-1.35)	0.898	0.929
	III	1.34 (1.01-1.76)	0.039	0.263
	IV	Reference	-	-
**Stroke**	I	1.29 (0.88-1.89)	0.188	0.423
	II	1.32 (0.90-1.93)	0.158	0.423
	III	1.18 (0.79-1.75)	0.426	0.707
	IV	Reference	-	-
**Ischemic Stroke**	I	1.18 (0.77-1.81)	0.445	0.707
	II	1.46 (0.95-2.23)	0.084	0.323
	III	1.09 (0.70-1.72)	0.693	0.850
	IV	Reference	-	-
**Hemorrhagic Stroke**	I	2.28 (0.92-5.63)	0.074	0.323
	II	1.04 (0.40-2.75)	0.929	0.929
	III	1.79 (0.70-4.58)	0.224	0.465
	IV	Reference	-	-
**PAD**	I	0.92 (0.64-1.32)	0.658	0.850
	II	0.64 (0.43-0.95)	0.025	0.227
	III	0.82 (0.56-1.19)	0.285	0.549
	IV	Reference	-	-
**CKD**	I	1.17 (0.93-1.49)	0.182	0.423
	II	0.84 (0.66-1.06)	0.149	0.423
	III	0.98 (0.77-1.25)	0.885	0.929
	IV	Reference	-	-
**Death**	I	0.94 (0.77-1.15)	0.544	0.816
	II	0.97 (0.79-1.18)	0.734	0.861
	III	0.96 (0.78-1.18)	0.685	0.850
	IV	Reference	-	-

Abbreviations: CHD, coronary heart disease; AF, atrial fibrillation; HF, heart failure; PAD, peripheral artery disease; CKD, chronic kidney disease; HR, hazard ratio; CI, confidence interval; FDR, false discovery rate; PFDR, Benjamini-Hochberg adjusted P-value.

Notes: FDR correction was applied using the Benjamini-Hochberg procedure across all subtype-outcome comparisons. All models were adjusted for age, sex, ethnicity, smoking status, alcohol consumption, systolic blood pressure, use of antihypertensive, glucose-lowering, and lipid-lowering medications, Townsend Deprivation Index, education level, and physical activity. Subtype IV served as the reference group (HR = 1.00 for all outcomes). P-values < 0.05 were considered statistically significant. PFDR indicate statistical significance after FDR correction (PFDR < 0.05).

Supplemental Table S12：Pairwise associations of metabolic subtypes with CKM-related outcomes within the MUO population.

Outcome	Comparison	HR (95% CI)	P-value
CHD	Subtype I vs Subtype II	0.92 (0.73–1.17)	0.509
	Subtype I vs Subtype III	0.96 (0.82–1.14)	0.669
	Subtype I vs Subtype IV	0.81 (0.67–0.99)	**0.044**
	Subtype II vs Subtype III	0.98 (0.79–1.21)	0.820
	Subtype II vs Subtype IV	0.98 (0.80–1.20)	0.843
	Subtype III vs Subtype IV	0.89 (0.71–1.11)	0.307
AF	Subtype I vs Subtype II	0.92 (0.73–1.18)	0.522
	Subtype I vs Subtype III	0.93 (0.79–1.10)	0.422
	Subtype I vs Subtype IV	1.29 (1.02–1.64)	**0.034**
	Subtype II vs Subtype III	1.04 (0.84–1.30)	0.700
	Subtype II vs Subtype IV	1.35 (1.07–1.70)	**0.012**
	Subtype III vs Subtype IV	1.36 (1.06–1.76)	**0.017**
HF	Subtype I vs Subtype II	1.15 (0.84–1.56)	0.380
	Subtype I vs Subtype III	0.77 (0.63–0.94)	**0.011**
	Subtype I vs Subtype IV	0.94 (0.69–1.27)	0.682
	Subtype II vs Subtype III	0.69 (0.53–0.89)	**0.005**
	Subtype II vs Subtype IV	1.02 (0.76–1.37)	0.909
	Subtype III vs Subtype IV	1.30 (0.96–1.77)	0.094
Stroke	Subtype I vs Subtype II	0.99 (0.65–1.50)	0.951
	Subtype I vs Subtype III	1.20 (0.89–1.62)	0.232
	Subtype I vs Subtype IV	1.29 (0.86–1.94)	0.219
	Subtype II vs Subtype III	1.06 (0.71–1.57)	0.771
	Subtype II vs Subtype IV	1.54 (1.03–2.31)	**0.035**
	Subtype III vs Subtype IV	1.00 (0.63–1.58)	0.998
Ischemic Stroke	Subtype I vs Subtype II	0.84 (0.53–1.33)	0.458
	Subtype I vs Subtype III	1.22 (0.87–1.72)	0.255
	Subtype I vs Subtype IV	1.15 (0.73–1.82)	0.540
	Subtype II vs Subtype III	1.22 (0.78–1.90)	0.374
	Subtype II vs Subtype IV	1.77 (1.13–2.75)	**0.012**
	Subtype III vs Subtype IV	0.90 (0.54–1.52)	0.705
Hemorrhagic Stroke	Subtype I vs Subtype II	2.75 (1.11–6.82)	**0.029**
	Subtype I vs Subtype III	1.27 (0.71–2.29)	0.425
	Subtype I vs Subtype IV	3.59 (1.37–9.40)	**0.009**
	Subtype II vs Subtype III	0.87 (0.36–2.13)	0.768
	Subtype II vs Subtype IV	1.01 (0.36–2.85)	0.983

Outcome	Comparison	HR (95% CI)	P-value
	Subtype III vs Subtype IV	1.76 (0.60–5.12)	0.301
**PAD**	Subtype I vs Subtype II	1.32 (0.85–2.06)	0.212
	Subtype I vs Subtype III	1.20 (0.90–1.59)	0.216
	Subtype I vs Subtype IV	0.91 (0.62–1.34)	0.640
	Subtype II vs Subtype III	0.75 (0.49–1.15)	0.189
	Subtype II vs Subtype IV	0.71 (0.47–1.08)	0.108
	Subtype III vs Subtype IV	0.95 (0.62–1.44)	0.794
**CKD**	Subtype I vs Subtype II	1.54 (1.19–1.99)	**<0.001**
	Subtype I vs Subtype III	1.21 (1.02–1.45)	**0.033**
	Subtype I vs Subtype IV	1.23 (0.95–1.60)	0.113
	Subtype II vs Subtype III	0.85 (0.67–1.08)	0.181
	Subtype II vs Subtype IV	0.85 (0.66–1.08)	0.189
	Subtype III vs Subtype IV	0.87 (0.66–1.15)	0.317
**Death**	Subtype I vs Subtype II	1.01 (0.80–1.27)	0.954
	Subtype I vs Subtype III	1.00 (0.85–1.16)	0.953
	Subtype I vs Subtype IV	0.85 (0.69–1.06)	0.146
	Subtype II vs Subtype III	0.91 (0.74–1.13)	0.393
	Subtype II vs Subtype IV	0.99 (0.80–1.22)	0.912
	Subtype III vs Subtype IV	0.94 (0.74–1.18)	0.579

Abbreviations: CHD, coronary heart disease; AF, atrial fibrillation; HF, heart failure; PAD, peripheral artery disease; CKD, chronic kidney disease; HR, hazard ratio; CI, confidence interval; MUO, metabolically unhealthy obesity.

Notes: Pairwise comparisons were performed among the four MUO subtypes using multivariable Cox proportional hazards models. For each outcome, participants with the corresponding prevalent disease at baseline were excluded. Models were adjusted for age, sex, ethnicity, smoking status, alcohol consumption, systolic blood pressure, use of antihypertensive, glucose-lowering, and lipid-lowering medications, Townsend Deprivation Index, education level, and physical activity. Each comparison is presented as the hazard ratio for the first-listed subtype relative to the second-listed subtype.

Supplemental Table S13: Fine-Gray subdistribution hazard models for CKM-related outcomes with all-cause death as competing risk.

	SHR（95%CI）P-value			
**Outcomes**	**Subtype I**	**Subtype II**	**Subtype III**	**Subtype IV**
**CHD**	1.16 (0.97-1.37)<br>0.099	1.33 (1.10-1.60)<br>0.003	1.21 (1.00-1.46)<br>0.044	1.36 (1.14-1.63)<br><0.001
**AF**	0.92 (0.77-1.09)<br>0.324	0.99 (0.82-1.19)<br>0.912	0.97 (0.80-1.16)<br>0.713	0.73 (0.60-0.90)<br>0.003
**HF**	1.10 (0.85-1.41)<br>0.479	1.13 (0.87-1.46)<br>0.363	1.43 (1.11-1.85)<br>0.005	1.07 (0.81-1.41)<br>0.627
**Stroke**	1.18 (0.86-1.62)<br>0.301	1.17 (0.83-1.64)<br>0.364	1.06 (0.76-1.49)<br>0.722	0.88 (0.61-1.27)<br>0.501
**Ischemic Stroke**	1.11 (0.78-1.58)<br>0.554	1.33 (0.91-1.94)<br>0.138	1.01 (0.69-1.47)<br>0.969	0.91 (0.60-1.37)<br>0.639
**Hemorrhagic Stroke**	1.44 (0.73-2.84)<br>0.295	0.58 (0.27-1.27)<br>0.176	1.09 (0.55-2.17)<br>0.798	0.58 (0.24-1.40)<br>0.228
**PAD**	1.02 (0.72-1.45)<br>0.901	0.70 (0.47-1.04)<br>0.077	0.85 (0.58-1.25)<br>0.413	1.14 (0.79-1.63)<br>0.485
**CKD**	1.54 (1.23-1.94)<br><0.001	1.11 (0.88-1.40)<br>0.389	1.27 (1.01-1.61)<br>0.044	1.32 (1.04-1.68)<br>0.021

Abbreviations: SHR, subdistribution hazard ratio ; CHD, coronary heart disease; AF, atrial fibrillation; HF, heart failure; PAD, peripheral artery disease; CKD, chronic renal failure; HR, hazard ratio; CI, confidence interval.

Notes: Data are presented as adjusted subdistribution hazard ratios (95% confidence intervals) with corresponding P values from Fine-Gray models treating all-cause death as a competing event. All models were adjusted for age, sex, ethnicity, smoking status, alcohol consumption, systolic blood pressure, use of antihypertensive, glucose-lowering, and lipid-lowering medications, Townsend Deprivation Index, education level, and physical activity. Non-MUO served as the reference group (HR = 1.00 for all outcomes). P-values < 0.05 were considered statistically significant.

Supplemental Table S14: Subtype × Metabolic syndrome component interaction analyses for each clinical outcome.

Outcome	Elevated WC	Elevated TG	Elevated BP	Elevated BG	Reduced HDL
**CHD**	0.086 (0.556)	0.949 (0.949)	0.632 (0.836)	0.159 (0.654)	0.538 (0.780)
**AF**	0.948 (0.949)	**0.024** (0.534)	0.915 (0.949)	0.795 (0.919)	0.825 (0.919)
**HF**	0.681 (0.851)	0.674 (0.851)	0.233 (0.654)	0.081 (0.556)	0.327 (0.691)
**Stroke**	0.623 (0.836)	0.858 (0.920)	0.224 (0.654)	0.724 (0.881)	0.128 (0.654)
**Ischemic stroke**	0.074 (0.556)	0.517 (0.780)	0.515 (0.780)	0.630 (0.836)	0.224 (0.654)
**Hemorrhagic stroke**	0.825 (0.919)	0.270 (0.676)	0.531 (0.780)	0.369 (0.691)	**0.042** (0.556)
**PAD**	0.496 (0.780)	0.133 (0.654)	0.229 (0.654)	0.303 (0.691)	0.061 (0.556)
**CKD**	0.489 (0.780)	0.366 (0.691)	0.345 (0.691)	0.177 (0.654)	0.268 (0.676)
**Death**	0.308 (0.691)	0.514 (0.780)	0.838 (0.919)	**0.022** (0.534)	0.221 (0.654)

Abbreviations: CHD, coronary heart disease; AF, atrial fibrillation; HF, heart failure; PAD, peripheral artery disease; CKD, chronic kidney disease; WC, waist circumference; TG, triglycerides; BP, blood pressure; BG, blood glucose; HDL, high-density lipoprotein.

Notes: Data are P for interaction (P_{FDR}). P values were derived from likelihood ratio tests comparing models with and without the Subtype × Component interaction term. P_{FDR} values were calculated using the Benjamini–Hochberg procedure across all 45 tests (global FDR). Bold indicates nominally significant interactions (P<0.05); none remained significant after FDR correction (all P_{FDR} >0.05). Similar results were obtained when FDR correction was applied by outcome or by component. All models were adjusted for age, sex, ethnicity, smoking status, alcohol consumption, systolic blood pressure, use of antihypertensive, glucose-lowering, and lipid-lowering medications, Townsend Deprivation Index, education level, and physical activity.

Supplemental Table S15: Sex-stratified hazard ratios for CHD and CKD by subtype, and sex-by-subtype interaction tests.

Outcomes	Sex	Subtype I	Subtype II	Subtype III	Subtype IV	P_interaction
**CHD**	Female	1.19 (0.76-1.87), P=0.454	1.54 (1.17-2.02), P=0.002	1.51 (1.01-2.26), P=0.042	1.27 (0.91-1.76), P=0.163	0.063
	Male	1.02 (0.83-1.25), P=0.848	1.06 (0.76-1.47), P=0.731	1.29 (1.01-1.63), P=0.039	1.11 (0.85-1.44), P=0.454	
**AF**	Female	0.77 (0.51-1.17), P=0.222	0.92 (0.72-1.18), P=0.506	0.71 (0.48-1.03), P=0.070	0.79 (0.57-1.10), P=0.161	0.993
	Male	0.92 (0.74-1.14), P=0.451	1.05 (0.74-1.47), P=0.792	0.99 (0.78-1.27), P=0.962	0.76 (0.55-1.06), P=0.105	
**HF**	Female	1.21 (0.70-2.09), P=0.496	1.39 (0.95-2.02), P=0.089	1.46 (0.89-2.39), P=0.138	1.19 (0.74-1.92), P=0.472	0.031
	Male	0.91 (0.67-1.23), P=0.538	0.58 (0.32-1.05), P=0.070	1.15 (0.82-1.60), P=0.413	1.22 (0.80-1.85), P=0.353	
**Stroke**	Female	1.29 (0.66-2.54), P=0.454	1.09 (0.70-1.69), P=0.697	1.09 (0.58-2.06), P=0.788	0.88 (0.48-1.62), P=0.689	0.674
	Male	1.09 (0.73-1.63), P=0.684	1.36 (0.74-2.51), P=0.324	1.04 (0.65-1.68), P=0.871	0.93 (0.51-1.67), P=0.802	
**Ischemic Stroke**	Female	1.13 (0.48-2.65), P=0.782	1.36 (0.82-2.26), P=0.229	1.49 (0.70-3.14), P=0.299	0.77 (0.38-1.60), P=0.487	0.554
	Male	1.01 (0.64-1.58), P=0.971	1.35 (0.70-2.63), P=0.372	0.93 (0.54-1.58), P=0.781	0.95 (0.50-1.80), P=0.877	
**Hemorrhagic Stroke**	Female	0.94 (0.31-2.90), P=0.917	0.27 (0.10-0.74), P=0.011	0.22 (0.06-0.78), P=0.019	0.80 (0.25-2.57), P=0.707	0.182
	Male	1.79 (0.68-4.75), P=0.239	2.09 (0.50-8.68), P=0.311	2.30 (0.79-6.72), P=0.127	0.39 (0.04-3.53), P=0.405	
**PAD**	Female	1.45 (0.72-2.92), P=0.300	0.82 (0.49-1.39), P=0.469	1.20 (0.60-2.39), P=0.605	1.40 (0.79-2.49), P=0.248	0.902
	Male	0.97 (0.62-1.50), P=0.875	0.82 (0.40-1.66), P=0.578	0.77 (0.47-1.26), P=0.294	0.97 (0.52-1.81), P=0.924	
**CKD**	Female	1.79 (1.20-2.69), P=0.005	1.23 (0.91-1.67), P=0.171	1.25 (0.83-1.88), P=0.282	1.36 (0.95-1.93), P=0.090	0.755
	Male	1.37 (1.00-1.86), P=0.048	1.00 (0.61-1.66), P=0.989	1.05 (0.75-1.48), P=0.761	1.54 (0.99-2.37), P=0.053	
**Death**	Female	1.15 (0.76-1.75), P=0.515	1.40 (1.07-1.83), P=0.015	1.22 (0.83-1.78), P=0.304	1.36 (0.97-1.91), P=0.071	0.319

Male	1.09 (0.87-1.36), P=0.465	0.96 (0.66-1.38), P=0.816	0.98 (0.76-1.26), P=0.897	1.44 (1.06-1.96), P=0.020

Abbreviations: CHD, coronary heart disease; CKD, chronic kidney disease; HR, hazard ratio; CI, confidence interval.

Notes: Data are presented as adjusted hazard ratios (95% confidence intervals) with corresponding P values from sex-stratified Cox models. All models were adjusted for age, ethnicity, smoking status, alcohol consumption, systolic blood pressure, glucose, use of antihypertensive, glucose-lowering, and lipid-lowering medications, Townsend Deprivation Index, education level, and physical activity. Non-MUO served as the reference group within each sex stratum. P_interaction values were derived from likelihood ratio tests comparing Cox models with and without Subtype × Sex interaction terms, adjusted for the same covariates plus sex as a main effect.

References

[1] Li Z, Yao Z, Liu Q. The association between white blood cell counts and metabolic health obesity among US adults[J/OL]. Frontiers in Nutrition, 2025, 12: 1458764. DOI:10.3389/fnut.2025.1458764.

[2] Jeong H R, Shim Y S, Lee H S, Hwang J S. Hemoglobin and hematocrit levels are positively associated with blood pressure in children and adolescents 10 to 18 years old[J/OL]. Scientific Reports, 2021, 11(1): 19052. DOI:10.1038/s41598-021-98472-0.

[3] Elmugabil A, Rayis D A, Abdelmageed R E, Adam I, Gasim G I. High level of hemoglobin, white blood cells and obesity among Sudanese women in early pregnancy: a cross-sectional study[J/OL]. Future science OA, 2017, 3(2): FSO182. DOI:10.4155/fsoa-2016-0096.

[4] Christakoudi S, Tsilidis K K, Evangelou E, Riboli E. Associations of obesity and body shape with erythrocyte and reticulocyte parameters in the UK Biobank cohort[J/OL]. BMC Endocrine Disorders, 2023, 23(1): 161. DOI:10.1186/s12902-023-01423-1.

[5] Kohsari M, Moradinazar M, Rahimi Z, Najafi F, Pasdar Y, Moradi A, Shakiba E. Association between RBC Indices, Anemia, and Obesity-Related Diseases Affected by Body Mass Index in Iranian Kurdish Population: Results from a Cohort Study in Western Iran[J/OL]. International Journal of Endocrinology, 2021, 2021: 1-13. DOI:10.1155/2021/9965728.

[6] Nkambule B B, Mxinwa V, Nyambuya T M, Dludla P V. The mean platelet volume and atherosclerotic cardiovascular-risk factors in adults with obesity: a systematic review and meta-analysis of observational studies[J/OL]. BMC Nutrition, 2022, 8(1): 47. DOI:10.1186/s40795-022-00541-8.

[7] Friedrich K, Sommer M, Strobel S, Thrum S, Blüher M, Wagner U, Rossol M. Perturbation of the Monocyte Compartment in Human Obesity[J/OL]. Frontiers in Immunology, 2019, 10: 1874. DOI:10.3389/fimmu.2019.01874.

[8] Gomez-Casado G, Jimenez-Gonzalez A, Rodriguez-Muñoz A, Tinahones F J, González-Mesa E, Murri M, Ortega-Gomez A. Neutrophils as indicators of obesity-associated inflammation: A systematic review and meta-analysis[J/OL]. Obesity Reviews: An Official Journal of the International Association for the Study of Obesity, 2025, 26(3): e13868. DOI:10.1111/obr.13868.

[9] Samocha-Bonet D, Justo D, Rogowski O, Saar N, Abu-Abeid S, Shenkerman G, Shapira I, Berliner S, Tomer A. Platelet counts and platelet activation markers in obese subjects[J/OL]. Mediators of Inflammation, 2008, 2008: 834153. DOI:10.1155/2008/834153.

[10] Liu F, Li Y, Li W, Feng R, Zhao H, Chen J, Du S, Ye W. The role of peripheral white blood cell counts in the association between central adiposity and glycemic status[J/OL]. Nutrition & Diabetes, 2024, 14(1): 30. DOI:10.1038/s41387-024-00271-9.

[11] Chen Z wen, Chen L ying, Dai H lei, Chen J hua, Fang L zheng. Relationship between alanine aminotransferase levels and metabolic syndrome in nonalcoholic fatty liver disease[J/OL]. Journal of Zhejiang University. Science. B, 2008, 9(8): 616-622. DOI:10.1631/jzus.B0720016.

[12] Tang Y, Fan Y, Su J, Yang Z, Liu Z. The association between serum albumin levels and metabolic syndrome based on the NHANES and two sample Mendelian randomization study[J/OL]. Scientific Reports, 2025, 15(1): 2861. DOI:10.1038/s41598-025-86859-2.

[13] Aqeel Rashid F, Mahdi S, Abd-Alkader Mahdy S, Thamer Salim A. Effect of Obesity on Plasma Alkaline Phosphatase Activity in Breast Cancer[J/OL]. Reports of Biochemistry & Molecular Biology, 2021, 10(2): 307-313. DOI:10.52547/rbmb.10.2.307.

[14] Ruan X, Li Z, Zhang Y, Yang L, Pan Y, Wang Z, Feng G S, Chen Y. Apolipoprotein A-I possesses an anti-obesity effect associated with increase of energy expenditure and up-regulation of UCP1 in brown fat[J/OL]. Journal of Cellular and Molecular Medicine, 2011, 15(4): 763-772. DOI:10.1111/j.1582-4934.2010.01045.x.

[15] Rajput-Williams J, Knott T J, Wallis S C, Sweetnam P, Yarnell J, Cox N, Bell G I, Miller N E, Scott J. Variation of apolipoprotein-B gene is associated with obesity, high blood cholesterol levels, and increased risk of coronary heart disease[J/OL]. Lancet, 1988, 2(8626-8627): 1442-1446. DOI:10.1016/s0140-6736(88)90930-0.

[16] Jalili V, Poorahmadi Z, Hasanpour Ardekanizadeh N, Gholamalizadeh M, Ajami M, Houshiarrad A, Hajipour A, Shafie F, Alizadeh A, Mokhtari Z, Shafaei H, Esmaeili M, Doaei S. The association between obesity with serum levels of liver enzymes, alanine aminotransferase, aspartate aminotransferase, alkaline phosphatase and gamma-glutamyl transferase in adult women[J/OL]. Endocrinology, Diabetes & Metabolism, 2022, 5(6): e367. DOI:10.1002/edm2.367.

[17] Cottam D R, Mattar S G, Barinas-Mitchell E, Eid G, Kuller L, Kelley D E, Schauer P R. The chronic inflammatory hypothesis for the morbidity associated with morbid obesity: implications and effects of weight loss[J/OL]. Obesity Surgery, 2004, 14(5): 589-600. DOI:10.1381/096089204323093345.

[18] Chen J M, Wu T Y, Wu Y F, Kuo K L. Association of the serum calcium level with metabolic syndrome and its components among adults in Taiwan[J/OL]. Archives of Endocrinology and Metabolism, 2023, 67(5): e000632. DOI:10.20945/2359-3997000000632.

[19] Vierhapper H, Nardi A, Grösser P. Prevalence of paradoxically normal serum cholestrol in morbidly obese women[J/OL]. Metabolism: Clinical and Experimental, 2000, 49(5): 607-610. DOI:10.1016/s0026-0495(00)80035-9.

[20] Gerchman F, Tong J, Utzschneider K M, Zraika S, Udayasankar J, McNeely M J, Carr D B, Leonetti D L, Young B A, de Boer I H, Boyko E J, Fujimoto W Y, Kahn S E. Body mass index is associated with increased creatinine clearance by a mechanism independent of body fat distribution[J/OL]. The Journal of Clinical Endocrinology and Metabolism, 2009, 94(10): 3781-3788. DOI:10.1210/jc.2008-2508.

[21] Jhatta C, Girdhar J, Gupta S, Verma I. To Compare the Level of Cystatin C in Type 2 Diabetes Mellitus with Obesity[J/OL]. Indian Journal of Endocrinology and Metabolism, 2020, 24(4): 312-318. DOI:10.4103/ijem.IJEM_408_20.

[22] Žiberna L, Jenko-Pražnikar Z, Petelin A. Serum Bilirubin Levels in Overweight and Obese Individuals: The Importance of Anti-Inflammatory and Antioxidant Responses[J/OL]. Antioxidants, 2021, 10(9): 1352. DOI:10.3390/antiox10091352.

[23] Jalili V, Poorahmadi Z, Hasanpour Ardekanizadeh N, Gholamalizadeh M, Ajami M, Houshiarrad A, Hajipour A, Shafie F, Alizadeh

A, Mokhtari Z, Shafaei H, Esmaeili M, Doaei S. The association between obesity with serum levels of liver enzymes, alanine aminotransferase, aspartate aminotransferase, alkaline phosphatase and gamma-glutamyl transferase in adult women[J/OL]. Endocrinology, Diabetes & Metabolism, 2022, 5(6): e367. DOI:10.1002/edm2.367.
[24] Par F, Sarvi F, Khodadost M, Pezeshki B, Doosti H, Tabrizi R. A Nonlinear Association of Body Mass Index and Fasting Blood Glucose: A Dose-Response Analysis From Fasa Adults Cohort Study (FACS)[J/OL]. Health Science Reports, 2025, 8(3): e70560. DOI:10.1002/hsr2.70560.
[25] Nam S Y, Lee E J, Kim K R, Cha B S, Song Y D, Lim S K, Lee H C, Huh K B. Effect of obesity on total and free insulin-like growth factor (IGF)-1, and their relationship to IGF-binding protein (BP)-1, IGFBP-2, IGFBP-3, insulin, and growth hormone[J/OL]. International Journal of Obesity and Related Metabolic Disorders: Journal of the International Association for the Study of Obesity, 1997, 21(5): 355-359. DOI:10.1038/sj.ijo.0800412.
[26] Bays H E, Kirkpatrick C F, Maki K C, Toth P P, Morgan R T, Tondt J, Christensen S M, Dixon D L, Jacobson T A. Obesity, dyslipidemia, and cardiovascular disease: A joint expert review from the Obesity Medicine Association and the National Lipid Association 2024[J/OL]. Journal of Clinical Lipidology, 2024, 18(3): e320-e350. DOI:10.1016/j.jacl.2024.04.001.
[27] Haksayar A, Donma M M, Batar B, Tepe B, Topçu B, Donma O. Association of Lipoprotein A rs10455872 Polymorphism with Childhood Obesity and Obesity-Related Outcomes[J/OL]. Diagnostics, 2025, 15(14): 1809. DOI:10.3390/diagnostics15141809.
[28] Mironov N, Haque M, Atfi A, Razzaque M S. Phosphate Dysregulation and Metabolic Syndrome[J/OL]. Nutrients, 2022, 14(21): 4477. DOI:10.3390/nu14214477.
[29] Huang R, Wang Y, Yan R, Ding B, Ma J. Sex Hormone Binding Globulin is an Independent Predictor for Insulin Resistance in Male Patients with Newly Diagnosed Type 2 Diabetes Mellitus[J/OL]. Diabetes Therapy: Research, Treatment and Education of Diabetes and Related Disorders, 2023, 14(10): 1627-1637. DOI:10.1007/s13300-023-01445-x.
[30] Liu L, Liu S, Song Q, Luo D, Su Y, Qi X, Wang Q, Ning J, Lv Y, Guan Q. Association of Metabolic Obesity Phenotypes and Total Testosterone in Chinese Male Population[J/OL]. Diabetes, Metabolic Syndrome and Obesity: Targets and Therapy, 2021, 14: 399-408. DOI:10.2147/DMSO.S293259.
[31] El-Eshmawy M M, Mahsoub N, Elsehely I. Serum total bilirubin is a risk factor of metabolic syndrome and its components in obese Egyptians[J/OL]. Porto Biomedical Journal, 2024, 9(6): 274. DOI:10.1097/j.pbj.0000000000000275.
[32] Timsans J, Palomäki A, Kauppi M. Gout and Hyperuricemia: A Narrative Review of Their Comorbidities and Clinical Implications[J/OL]. Journal of Clinical Medicine, 2024, 13(24): 7616. DOI:10.3390/jcm13247616.
[33] Zeng R X, Xu J P, Zhang Y Z, Tan J W, Kong Y J, Zhang M Z, Guo L H. Associations of total protein, albumin, and globulin with insulin resistance: an NHANES study[J/OL]. Frontiers in Endocrinology, 2024, 15: 1393137. DOI:10.3389/fendo.2024.1393137.
[34] Kjaergaard A D, Teumer A, Witte D R, Stanzick K J, Winkler T W, Burgess S, Ellervik C. Obesity and Kidney Function: A Two-Sample Mendelian Randomization Study[J/OL]. Clinical Chemistry, 2022, 68(3): 461-472. DOI:10.1093/clinchem/hvab249.
[35] Vanlint S. Vitamin D and obesity[J/OL]. Nutrients, 2013, 5(3): 949-956. DOI:10.3390/nu5030949.
